# Advancing multifunctional carbon fibre composites: the role of nanomaterials in boosting electrochemical performance for energy storage

**DOI:** 10.1098/rsos.250606

**Published:** 2025-08-20

**Authors:** Farag M. A. Altalbawy, Ayad Abdulrazzaq Mutar, Ramdevsinh Jhala, Nagaraj Patil, Fadhil Faez Sead, Debasish Shit, V. K. Bupesh Raja, Abinash Mahapatro, Jamal K. Abbas, Hadi Noori

**Affiliations:** ^1^ Department of Chemistry, University of Tabuk, Tabuk, Saudi Arabia; ^2^ Medical Laboratory Techniques Department, College of Health and Medical Technology, Al-maarif University, Anbar, Iraq; ^3^ Marwadi University Research Center, Department of Mechanical Engineering, Faculty of Engineering & Technology Marwadi University, Rajkot-360003, Rajkot, Gujarat, India; ^4^ Department of Mechanical Engineering, School of Engineering and Technology, JAIN (Deemed-to-be University), Bengaluru, Karnataka, India; ^5^ Department of Dentistry, College of Dentistry, The Islamic University, Najaf, Iraq; ^6^ Department of Medical Analysis, Medical Laboratory Technique College, the Islamic University of Al Diwaniyah, Al Diwaniyah, Iraq; ^7^ Department of Medical Analysis, Medical Laboratory Technique College, the Islamic University of Babylon, Babylon, Iraq; ^8^ Centre for Research Impact & Outcome, Chitkara University Institute of Engineering and Technology, Chitkara University, Rajpura, 140401, Rajpura, Punjab, India; ^9^ Department of Mechanical Engineering, Sathyabama Institute of Science and Technology, Chennai, Tamil Nadu, India; ^10^ Department of Mechanical Engineering, Siksha 'O' Anusandhan (Deemed to be University), Bhubaneswar, Odisha, India; ^11^ Al-Nisour University College, Baghdad, Iraq; ^12^ Department of Chemistry, Young Researchers and Elite Club, Tehran, Iran

**Keywords:** CFCs, nanomaterials, energy harvesting, surface functionalization, shape morphing, charge transfer

## Abstract

Carbon fibre composites (CFCs) hold significant promise for energy storage and harvesting applications owing to their exceptional strength-to-weight ratio and structural versatility, but their electrochemical performance is constrained by inherent limitations such as low surface area and restricted ion transport pathways. This review examines how strategic integration of nanomaterials—including graphene, carbon nanotubes and MXenes—can overcome these challenges by enhancing surface reactivity, improving electrical conductivity and facilitating efficient ion diffusion, thereby enabling high-performance multifunctional composites. We discuss key advances in nanomaterial-incorporated CFCs for structural batteries and supercapacitors, where tailored interfaces and hierarchical architectures contribute to superior energy and power densities, as well as their emerging role in integrated energy harvesting systems that combine energy storage with triboelectric, piezoelectric or thermoelectric conversion capabilities. The analysis further addresses critical manufacturing challenges related to nanomaterial dispersion, interfacial bonding and scalable processing, while evaluating solutions such as advanced deposition techniques and hybrid material designs. By systematically reviewing both fundamental mechanisms and practical considerations, this work provides insights into the development of next-generation smart composites that simultaneously achieve mechanical robustness and advanced electrochemical functionality for applications ranging from wearable electronics to electric vehicles and aerospace systems.

## 1. Introduction

The rapid development of advanced technologies, particularly in the energy sector, is driving the demand for new materials that exhibit multifunctionality, combining mechanical robustness with excellent electrochemical performance. Carbon fibre composites (CFCs) have emerged as one of the most promising materials for a wide range of applications, including aerospace, automotive and renewable energy systems. This popularity is largely owing to the unique properties of carbon fibres (CFs), such as high tensile strength, low weight, excellent electrical conductivity and high resistance to corrosion [1–4]. These characteristics make CFCs ideal candidates for applications where both structural integrity and electrical performance are crucial. However, despite their inherent advantages, the electrochemical efficiency of CFs remains limited when used in standalone applications like energy storage systems. Their low surface area, limited active sites for ion adsorption and insufficient ion transport capacity restrict their ability to function efficiently in devices such as supercapacitors and batteries. To address these challenges, researchers have increasingly focused on enhancing the electrochemical properties of CFCs by integrating nanomaterials [5–7]. Nanomaterials, such as carbon nanotubes (CNTs), graphene, metal oxides and conducting polymers, offer several advantages, including a higher surface area, improved charge transfer rates and additional active sites for ion storage. These properties directly impact the performance of electrochemical systems, leading to higher energy densities, faster charge/discharge rates and better cycling stability. As a result, nanomaterial-modified CFCs are gaining attention for their potential to transform various energy storages and harvesting technologies, particularly in applications requiring lightweight, durable and energy-efficient materials [8–10].

Energy storage systems, such as lithium-ion (Li-ion) batteries, structural batteries (SBs) and supercapacitors, are critical for the transition to sustainable energy. CFCs, with their high conductivity and mechanical strength, are uniquely positioned to serve in these systems, where energy storage components must also bear structural loads. SBs, which combine the roles of energy storage and mechanical support within a single component, are one example of how CFCs can enhance the efficiency and versatility of energy storage technologies. SBs are multifunctional energy storage systems that integrate electrochemical energy storage with load-bearing capabilities, enabling a single material to simultaneously store energy and serve as a structural component, thus reducing overall system weight and enhancing efficiency [9].

These systems are particularly attractive for use in electric vehicles (EVs), where reducing weight and maximizing energy storage capacity are paramount. The introduction of nanomaterials into CFCs has been shown to significantly enhance their electrochemical performance in these applications. Graphene, for example, improves the electrical conductivity and mechanical strength of CFCs while increasing their surface area, thereby facilitating faster electron transfer and better ion transport [11,12]. CNTs, which have exceptional electrical and mechanical properties, can be integrated into the composite matrix to create more conductive networks, improve structural integrity and increase the available surface area for electrochemical reactions. In addition to carbon-based nanomaterials, metal oxides like MnO₂ and TiO₂ have also been explored for their ability to enhance the charge storage capabilities of CFCs. These metal oxides provide high specific capacitance and improve the cycling durability of energy storage devices [13].

Energy harvesting is another emerging field where CFCs hold significant promise. Energy harvesting technologies capture ambient energy, such as mechanical vibrations, heat or light, and convert it into usable electrical energy. This approach is increasingly being used to power small-scale devices, sensors and wireless systems, offering a sustainable alternative to conventional battery-powered systems. CFCs are particularly well-suited for energy harvesting applications owing to their high mechanical strength, flexibility and electrical conductivity. These properties enable them to function as both structural reinforcements and active components in energy harvesting devices. In particular, the combination of CFCs and nanomaterials has opened new possibilities for enhancing the efficiency of energy harvesting systems. By introducing nanomaterials, the electrochemical properties of CF are improved, enabling more efficient energy conversion processes [5,10]. For example, triboelectric nanogenerators (TENGs), which harvest mechanical energy through the triboelectric effect, can benefit from the high surface area and conductivity of CFCs modified with nanomaterials. Similarly, piezoelectric and thermoelectric systems, which convert mechanical stress and temperature differences into electrical energy, can be optimized by integrating nanomaterials into the CF matrix, improving both mechanical durability and energy conversion efficiency [11].

One of the primary challenges in developing nanomaterial-modified CFCs for energy storage and harvesting applications is achieving uniform dispersion and alignment of nanomaterials within the composite matrix. Advanced manufacturing techniques, such as chemical vapour deposition (CVD), resin transfer moulding (RTM) and surface functionalization, have been developed to address these challenges. These methods allow for precise control over the distribution and orientation of nanomaterials within the CF matrix, ensuring optimal performance in electrochemical systems. For instance, surface functionalization techniques, such as the introduction of hydroxyl, carboxyl or epoxy groups, can enhance the wettability and surface charge density of CFs, improving their interaction with electrolytes in energy storage systems. Similarly, chemical doping and plasma treatment can be used to tailor the surface chemistry of CFs to achieve specific electrochemical properties, such as increased charge storage capacity and enhanced ion adsorption. Despite the significant progress that has been made in the development of nanomaterial-modified CFCs, several challenges remain. One of the main obstacles is the high cost of production, which is primarily owing to the complex manufacturing processes required to achieve uniform nanomaterial dispersion and alignment [14,15]. Additionally, long-term stability in harsh electrochemical environments, such as high temperatures, corrosive conditions and repeated mechanical stress, is a critical concern for the widespread adoption of these materials in commercial applications. To address these issues, researchers are exploring strategies such as hybrid nanomaterial composites, which combine multiple types of nanomaterials to achieve synergistic effects, and scalable manufacturing processes that reduce production costs while maintaining high performance [4,8].

In addition to their potential for energy storage and harvesting, CFCs have shown promise in multifunctional applications, such as structural health monitoring and shape-morphing devices. The ability to combine energy storage with mechanical functionality in a single material system opens new possibilities for smart structures that can sense and respond to their environment. For example, shape-morphing composites, which can change shape in response to external stimuli such as electrical or thermal inputs, could be integrated with energy storage systems to create self-powered adaptive structures. Shape-morphing composites are smart materials designed to undergo controlled, reversible shape changes in response to external stimuli, such as electrical, thermal or mechanical inputs, enabling adaptive functionalities for applications in dynamic structures and devices [11,15]. These materials could be used in a wide range of applications, from aerospace and automotive components to wearable devices and biomedical implants [11,15].

This review explores the electrochemical properties of CFCs and the role of nanomaterials in enhancing their performance for energy storage and harvesting applications. It also highlights the latest advancements in manufacturing techniques, the challenges associated with scaling up production, and the potential future directions for these multifunctional materials. By integrating nanomaterials with CFCs, researchers are unlocking new possibilities for improving the energy density, charge/discharge rates and cycling durability of energy storage systems while also paving the way for innovative energy harvesting technologies.

### 1.1. Literature review methodology

This review was conducted using a systematic approach to identify and evaluate relevant literature on CFCs enhanced with nanomaterials for energy storage and harvesting applications. The literature search was performed across multiple academic databases, including Scopus, Web of Science, PubMed and Google Scholar, to ensure comprehensive coverage of peer-reviewed articles, conference proceedings and book chapters published between 2010 and 2025. The following keywords and their combinations were used: ‘carbon fiber composites’, ‘nanomaterials’, ‘graphene’, ‘carbon nanotubes’, ‘MXenes’, ‘energy storage’, ‘energy harvesting’, ‘structural batteries’, ‘supercapacitors’, ‘triboelectric’, ‘piezoelectric’, ‘thermoelectric’, ‘electrochemical performance’, ‘surface functionalization’ and ‘manufacturing challenges.’

Inclusion criteria were defined to select studies that: (i) focused on CFCs integrated with nanomaterials for electrochemical applications, (ii) reported experimental or theoretical data on energy storage or harvesting performance, (iii) were published in English, and (iv) provided insights into material synthesis, characterization or practical applications. Exclusion criteria included studies lacking primary data, non-peer-reviewed sources or those unrelated to electrochemical applications of CFCs. The initial search yielded approximately 500 articles, which were screened based on titles and abstracts. After removing duplicates and irrelevant studies, 182 articles were selected for full-text review, and 154 were ultimately included in this manuscript based on their relevance and quality. The selection process was conducted independently by two authors, with discrepancies resolved through discussion to ensure consistency. This methodology ensured a robust and representative analysis of the current state of the field.

## 2. Carbon fibre electrochemical properties

CFs possess unique electrochemical properties, making them highly suitable for a wide range of energy storage devices, including supercapacitors, batteries and SBs. These properties stem from their high electrical conductivity, large surface area and excellent mechanical strength, which together enhance their performance as electrodes in electrochemical systems. The electrochemical behaviour of CF is influenced by several factors, including surface chemistry, morphology and specific ion interactions within their structure. These characteristics make CF an attractive material for multifunctional systems that require both mechanical and electrochemical functionalities. The electrical conductivity of CF plays a crucial role in their electrochemical performance, particularly in energy storage applications such as batteries and supercapacitors. CFs exhibit high conductivity due to their sp² hybridized carbon atoms arranged in a graphitic structure, enabling efficient electron transport. This structure consists of graphene-like layers, where each carbon atom forms strong covalent bonds with three neighbours while the remaining electron forms a delocalized pi bond that facilitates free electron movement. Additionally, CFs’ fibre morphology, consisting of long, continuous filaments, allows electrons to travel over large distances with minimal resistance [16,17]. Table 1 shows the structural, thermal and electrical characteristics of CF in electrochemistry [16–19].

**Table 1.** Structural, thermal and electrical characteristics of carbon fibre in electrochemistry.

property	description	relevance to electrochemistry
density	1.6–2.0 g cm^−3^	low density contributes to lightweight electrochemical devices
tensile strength	3500–7000 MPa	high strength enables the fabrication of robust electrodes for energy storage
Young’s modulus (elastic modulus)	230–500 GPa	high rigidity supports structural stability in electrochemical systems
electrical conductivity	high, 10³ to 10⁶ S m^−1^	essential for efficient electron transfer in electrochemical reactions
thermal conductivity	10–40 W (m·K)^−1^	ensures heat dissipation during electrochemical reactions
electrochemical stability	high resistance to corrosion and oxidation	enhances durability in electrochemical devices, such as batteries and sensors
surface area	typically 1–5 m² g^−1^ for activated CFs	increased surface area provides more active sites for reactions in energy storage and sensors
capacitance	moderate capacitance for activated carbon-based fibres	suitable for use in supercapacitors and energy storage devices
porosity	high surface porosity with micro and mesopores	promotes ion transport, crucial for supercapacitors and batteries
corrosion resistance	very high resistance to corrosion, particularly in acidic or basic environments	enhances long-term performance in electrochemical cells
fatigue resistance	high fatigue resistance after repeated stress	ensures longevity of materials in cyclic electrochemical applications
moisture absorption	low moisture absorption, around 0.05–0.1% by weight	minimizes performance degradation due to environmental humidity
surface chemistry	can be modified for various functional groups (e.g. hydrophilic, hydrophobic)	functionalization improves charge storage capacity and electrochemical reactivity
processing temperature	can withstand temperatures up to 3000°C for high-performance varieties	suitable for high-temperature electrochemical processes

Its excellent electrical conductivity (ranging from 10³ to 10⁶ S m^−1^) enables efficient electron transfer in electrochemical reactions, while its high surface area (1–5 m² g^−1^ for activated CFs) provides abundant active sites for these reactions. Additionally, CFs’ high porosity, with micro and mesopores, promotes ion transport, which is crucial for the performance of supercapacitors and batteries. The material’s high electrochemical stability, including resistance to oxidation and corrosion, ensures that it can withstand harsh electrochemical environments, making it a durable choice for long-term applications. Furthermore, CF offers impressive mechanical properties, such as high tensile strength (3500–7000 MPa) and fatigue resistance, which provide structural integrity to electrochemical devices. Its ability to resist corrosion in both acidic and basic environments adds to its longevity and reliability in sensors and energy storage devices. The material’s low moisture absorption prevents degradation in humid conditions, and its ability to be surface-functionalized (e.g. hydrophilic or hydrophobic modifications) enhances its electrochemical reactivity. Together, these properties make CF an ideal material for a wide range of electrochemical applications, from batteries to supercapacitors and sensors.

**Figure 1.**
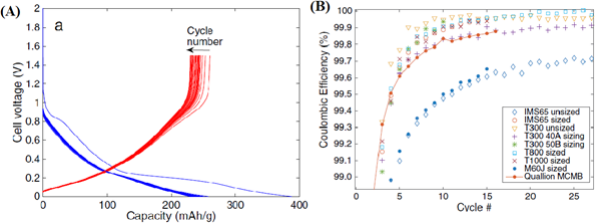
(A) Galvanostatic charge/discharge curves for T800 fibre and (B) coulombic efficiency versus cycle number for different carbon fibres compared with Quallion MCMB commercial graphite electrode material (solid line). Reproduced with permission from Hagberg *et al.* [20]. © ECS.

Figure 1 presents representative galvanostatic cycling results for modulus CFs, such as T800 fibre along with the coulombic efficiency of various CFs. Figure 1A shows the galvanostatic charge/discharge curves for T800 CF, illustrating its electrochemical performance during Li-ion insertion and extraction. The *x*-axis represents the capacity (mAh g^−1^), indicating how much charge the fibre can store, while the *y*-axis shows the voltage (V) during the process. The curves exhibit plateaus, which correspond to specific electrochemical reactions as lithium ions (Li^+^) are inserted into or extracted from the fibre. The T800 fibre demonstrates a high capacity of around 350 mAh g^−1^, comparable with commercial graphite electrodes, and the overlapping charge/discharge curves indicate excellent reversibility. This means the fibre can efficiently store and release Li^+^ without significant degradation, making it a promising material for battery applications. Figure 1B compares the coulombic efficiency of different CFs, including T800, over multiple charge/discharge cycles. Coulombic efficiency, plotted on the *y*-axis, measures the reversibility of the electrochemical process, with values close to 100% indicating minimal energy loss. The *x*-axis represents the cycle number, showing how the efficiency changes over time. The T800 fibre maintains a high coulombic efficiency (close to or above 99%), similar to or better than commercial graphite electrodes, demonstrating its stability and durability during cycling. This high efficiency, combined with the fibre’s high capacity, highlights its potential as a reliable electrode material for Li-ion batteries, though mechanical property changes during cycling remain a consideration.

In energy storage systems, high conductivity is essential for fast charge and discharge cycles. CFs form conductive networks within composites, making them suitable for SBs and supercapacitors, where both mechanical strength and electrochemical performance are required. Their electrochemical properties can be further enhanced through surface modifications, such as introducing functional groups like hydroxyl, carboxyl or epoxy groups, which improve wettability, surface charge density and ion adsorption capacity. Techniques like oxidation, plasma treatment and chemical doping tailor the surface properties of CFs for specific energy storage applications. The morphology of CFs significantly impacts their electrochemical performance. Their hierarchical structure, which includes microporous and mesoporous features, provides additional surface area for ion storage, boosting charge capacity [17,18]. The pore size distribution within CFs determines how efficiently ions can be inserted and removed during charge–discharge cycles, affecting the rate capability and efficiency of energy storage systems. Techniques to control CF porosity enable further optimization of their electrochemical properties.

CFs' high conductivity ensures efficient electron flow, reducing internal resistance and enhancing device performance in batteries and supercapacitors. Their ability to form a three-dimensional conductive network within composite electrodes allows for uniform electron transport, particularly crucial for SBs where electrodes are integrated into the structural material. In such systems, CFs not only enhances electrical conductivity but also provide mechanical strength, enabling materials to bear mechanical loads while storing and delivering energy. The conductive network reduces electrical resistance, leading to efficient energy storage and release, particularly in high-performance applications like EVs, where both structural integrity and electrochemical performance must be optimized. CFs' high electrical conductivity also benefits multifunctional materials such as SBs, where they provide both electrochemical functionality and mechanical strength [17–19].

CF-based anodes and cathodes must function synergistically to ensure efficient ion flow and minimal resistance. Since CFs are often used for both anode and cathode electrodes in SBs, balancing electrochemical and mechanical properties is essential. Effective electrode design and material integration are key to developing high-performance structural composites for energy storage and load-bearing applications in EVs and aerospace. In SBs, the interface between CF electrodes and the electrolyte is critical for minimizing internal resistance and ensuring efficient charge transfer. Ionic conductivity and electrochemical stability under high charge–discharge cycles must be carefully optimized to ensure long-term efficiency and stability. By tailoring surface chemistry, morphology and coating materials, CF-based electrodes can meet the demands of advanced energy storage systems. As research in this field progresses, CFs hold significant promise for the development of high-performance, sustainable and lightweight energy storage solutions [19,20].

## 3. Synthesis methods used for carbon fibre composites

The fabrication of CFCs uses a variety of techniques that differ in cost, scalability and mechanical performance. These methods can be grouped into manual, mould-based, continuous and advanced techniques. Manual techniques, including hand lay-up and wet lay-up, are commonly used for prototyping or low-volume custom parts owing to their simplicity and low cost. Layers of CF are manually impregnated with resin (typically epoxy, polyester or vinyl ester) and cured at room or elevated temperature. While these methods offer flexibility in shape, they suffer from inconsistent fibre wetting and high void content [21,22]. Vacuum-assisted and closed-mould techniques like vacuum bagging and RTM improve fibre-resin interaction by applying pressure or vacuum. Vacuum bagging involves sealing the laminate under a plastic film and applying vacuum (60–90 kPa), enhancing impregnation and reducing voids [23]. RTM injects resin into a closed mould containing dry carbon preforms under moderate pressure (2–10 MPa), offering better dimensional control and uniform quality [24].

High-performance thermal-pressure methods, such as autoclave and compression moulding, are used when high structural integrity is needed. Autoclave moulding cures prepreg layers under both heat (120−180°C) and pressure (5–10 bar), yielding low-void, aerospace-grade parts [25]. Compression moulding involves placing a resin-infused preform in a heated mould, followed by high pressure (20–150 MPa) to shape and cure the part quickly and consistently [26]. Continuous and automated processes include filament winding and pultrusion. Filament winding winds resin-impregnated fibres around a rotating mandrel at defined angles (30°−90°) to create cylindrical structures like tanks and pipes [27]. Pultrusion draws resin-saturated rovings through a heated die (150−200°C), allowing continuous production of high-strength, uniform cross-section profiles [28]. Advanced techniques such as additive manufacturing (three-dimensional printing), co-electrospinning, and CVD are expanding the versatility of CFCs. Additive manufacturing combines CF with thermoplastics (e.g. polylactic acid (PLA), nylon) for custom, layer-by-layer parts [29]. Co-electrospinning uses high-voltage jets to fabricate nanofibres with tunable properties [30]. CVD enhances surface conductivity by depositing carbonaceous coatings (e.g. graphene) on CF surfaces at elevated temperatures (500−1200°C) [31]. Each method offers distinct advantages depending on production volume, geometry complexity and performance requirements. Details of the synthesis methods and their comparative table (table 2) are provided in the electronic supplementary material, appendix.

**Table 2.** Comparison of manufacturing methods for CFCs: characteristics, advantages and applications.

method	process type	resin type	typical applications	cost	cycle time	production volume	main advantages	main disadvantages	ref
hand lay-up method	manual	epoxy, polyester, vinyl ester	low-volume, custom parts	low	long	low	simple, low cost, flexible for complex shapes	labour-intensive, inconsistent quality, high void content	[21]
vacuum bagging	manual + vacuum	epoxy, polyester, vinyl ester	aerospace, automotive, marine	moderate	medium to long	low to medium	reduced void content, improved mechanical properties	requires skilled labour, longer processing time, potential for resin wastage	[23]
RTM	semi-automated	epoxy, polyester, vinyl ester	automotive, aerospace, industrial parts	moderate to high	medium	medium	high-quality, good for complex shapes, low void content	high tooling cost, resin flow issues, limited to certain fibre architectures	[24]
filament winding	automated	epoxy, vinyl ester, polyester	aerospace, pressure vessels, pipes	moderate	medium to long	medium to high	high strength, continuous fibres, customizable profiles	limited to cylindrical or rotationally symmetric parts, high setup cost	[27]
pultrusion	continuous	epoxy, polyester, vinyl ester	structural components, beams, rods	moderate to high	fast	high	continuous production, uniform profile, high fibre content	limited to constant cross-sectional profiles, high initial equipment cost	[28]
autoclave moulding	high pressure + heat	epoxy, vinyl ester, phenolic	aerospace, automotive, high-performance parts	high	long	low to medium	high-quality, low voids, excellent mechanical properties	expensive equipment, long cycle times, high energy consumption	[25]
compression molding	high pressure + heat	epoxy, vinyl ester, phenolic	automotive, electrical enclosures, consumer goods	moderate to high	medium to short	medium to high	shorter cycle times, low void content, high repeatability	high tooling cost, limited to relatively simple geometries	[26]
additive manufacturing (three-dimensional printing)	layer-by-layer	nylon, PLA, epoxy, polycarbonate	prototyping, automotive, aerospace, medical	moderate to high	very short to medium	low to medium	custom geometries, rapid prototyping, low-volume production	lower mechanical strength, anisotropic properties, limited material selection	[29]
wet lay-up	manual	epoxy, polyester, vinyl ester	marine, low-volume, prototype parts	low	long	low	low cost, flexible, good for custom parts, simple process	high resin content, poor repeatability, high void fraction	[22]
co-electrospinning	electrospinning	polymers (nylon, PLA, epoxy)	biomedical, filtration, sensors	moderate to high	medium	low	tailored fibre structures, multi-material composites	difficult to scale, requires precise process control, material limitations	[30]
CVD	vapour-phase deposition	carbon precursors (e.g. acetylene)	electronics, aerospace, advanced composites	high	medium to long	low to medium	high-quality coatings, controlled surface properties	high cost, complex process, requires specialized equipment	[31]

## 4. Advancing energy harvesting with carbon fibre composites

CFs, known for their high tensile strength, flexibility and low weight, have gained attention for multifunctional energy-harvesting applications [32]. Their mechanical resilience enables efficient conversion of ambient energy sources—particularly vibrations and motion—into electricity through piezoelectric and electrochemical mechanisms. When subjected to mechanical stress, CFs exhibits a piezoelectric-like response, generating electrical charges owing to deformation-induced polarization. This is further enhanced by electrochemical effects such as ion intercalation, resulting in a synergistic energy conversion process [33]. In dynamic environments, CFs integrated into structural systems can harvest mechanical energy with notable efficiency. Xie *et al.* demonstrated that CF-based composites could capture up to 25% of mechanical energy from bending-twisting oscillations, maintaining performance over 1000 cycles. Their integration into wearable devices enabled energy densities of 18 nW g^−1^, suitable for powering low-energy sensors [34].

Beyond mechanical harvesting, CFs also offer promise in thermal energy capture. Zhao *et al.* developed mineral-impregnated CF grids with thermoelectric properties, achieving 1.8 mV and 22.3 nW under a 50 K gradient. This thermal-to-electrical conversion (efficiency: 3.2%) highlights the feasibility of embedding CF-based thermoelectric elements in building materials for passive energy generation [35]. Together, these findings underscore the potential of CF-based composites in self-powered, multifunctional systems for structural health monitoring, wearable electronics and energy-smart infrastructure.

## 5. Mechanisms of energy harvesting using carbon fibre composites

### 5.1. Triboelectric energy harvesting

Triboelectric energy harvesting (TEH) uses the contact electrification effect, where mechanical motion between dissimilar materials generates static charges. CFCs, known for their high conductivity, flexibility, and surface area serve as both structural and active components in TENGs, making them ideal for harvesting mechanical energy from vibrations, bending or compression [36,37]. When paired with triboelectric materials like polytetrafluoroethylene (PTFE) or polydimethylsiloxane (PDMS), CFCs enable efficient charge generation through friction and separation. Recent studies have reported output voltages up to 230 V and current densities of 4.5 μA cm^−2^ in CFC-based TENGs, demonstrating their capability for powering small electronics [38,39]. Additionally, their mechanical durability allows for stable performance over thousands of cycles, with energy conversion efficiencies exceeding 15% in optimized designs [40].

Surface functionalization is a key strategy for enhancing triboelectric performance. Functional groups such as hydroxyl, carboxyl or carbonyl introduced via chemical or plasma treatments improve surface polarity and charge affinity. Functionalized CFCs have achieved power densities of 250 μW cm^−2^, while hybridization with graphene oxide (GO) has increased this to 320 μW cm^−2^, ideal for wearable applications [39,41]. Advanced interfacial engineering, including the use of modified epoxy matrices and ionic conductive adhesives, has minimized charge leakage and enhanced durability. For instance, interfacial optimization led to consistent output voltage of 200 V and power density of 2.1 W m^−2^ after 50 000 cycles [41]. Architectural innovations—such as sandwich structures and woven laminates—have further improved contact efficiency, with peak outputs of 1.2 W m^−2^ in stress-responsive systems [38,39]. The CFC-TENG integrates CF paper with PTFE layers and Ecoflex substrates. It features high mechanical flexibility, hydrophobicity and environmental resistance. Raman and scanning electron microscope analyses confirm the graphitic nature and fibrous network of the carbon paper, contributing to high surface charge retention and robustness under mechanical deformation [42].

Integration with energy storage devices extends the functionality of CFC-TENGs. Hybrid systems combining TENGs with MXene-modified CFs and Zn-Cu selenide nanostructures have achieved capacitance of 120 F g^−1^ and energy density of 35 Wh kg^−1^ [5]. Moreover, bifunctional CFs embedded in flexible TENGs generated 0.5 mW cm^−2^, charged capacitors to 250 mV and powered 38 LEDs while withstanding 12 000 cycles [42]. Future directions include additive manufacturing, green fibre precursors, and solid-state ionic media to enhance efficiency and sustainability. Arch-shaped carbon fiber reinforced polymer (CFRP) hybrids have reached over 1.5 W m^−2^, and TEH systems integrated into structural elements show promise for powering autonomous sensors and flexible electronics in real-time environments [43].

### 5.2. Thermoelectric energy harvesting

The thermoelectric effect enables conversion between thermal gradients and electric voltage, offering a promising approach for waste heat recovery. CFCs, combining high electrical conductivity and low density, have emerged as structurally robust candidates for thermoelectric generators (TEGs) [33,44]. Key parameters such as Seebeck coefficient, electrical conductivity and thermal conductivity directly influence their thermoelectric performance. Aligned CFs enhance directional conductivity, while polymer matrices help maintain thermal gradients but may hinder charge transport. Laminated TEG-enabled CFCs have demonstrated power densities up to 25 μW cm^−2^ under 60−75°C gradients using M40B and A38 CF tows with Seebeck coefficients of +33.85 and −11.83 μV K^−1^, respectively [45]. Surface modifications, including chemical doping and polymer coatings, have improved figure of merit (ZT) by 20%, enhancing Seebeck coefficients to 45−55 μV K^−1^ and reducing thermal conductivity to 0.25 W (m·K)^−1^ [46,47].

**Figure 2.**
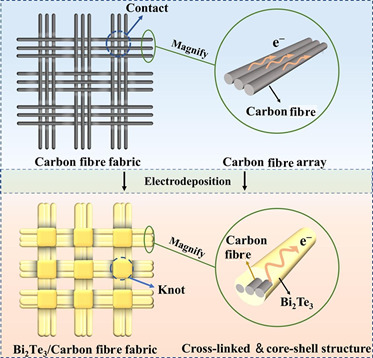
Modification of carbon fibre fabric for flexible thermoelectric energy harvesting. Reproduced with permission from [47]. © 2023 Elsevier.

Figure 2 illustrates the modification of CF fabric for flexible thermoelectric energy conversion. The process involves creating a cross-linked and core-shell structure using CF fabric, which is enhanced with materials like bismuth telluride (BiTe) to improve thermoelectric properties. The thermoelectric effect is used to convert temperature gradients into electrical energy, enabling energy harvesting from waste heat. This modification aims to optimize the fabric’s ability to generate power in flexible applications, making it suitable for wearable technology and other energy-harvesting devices. The electrodeposition technique is employed to ensure a uniform and efficient integration of thermoelectric materials onto the CF array, enhancing the overall performance of the energy conversion process [47].

These modifications also yield mechanical flexibility, essential for wearable devices. Flexible thermoelectric CFCs incorporating Bi₂Te₃ nanostructures have reached power factors of 120 μW (m·K²)^−1^ [47], while electrodeposited films on CF arrays ensure uniform material distribution. Recycled CFs, chemically intercalated and doped, achieved a 1.74-fold Seebeck improvement and power factor of 80 μW (m·K²)^−1^, highlighting sustainability potential [48]. Porosity control in cement-based expanded graphite/carbon fiber cement composites (EG-CFRCs) has improved phonon scattering and ZT values, though excessive porosity can reduce electrical conductivity [48].

**Figure 3.**
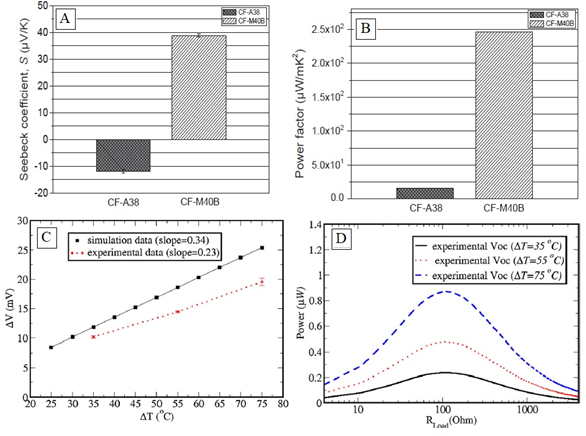
(A) Seebeck coefficient and (B) power factor values of CF-A38 and CF-M40B tows; (C) voltage output (Δ*V*) versus temperature difference (Δ*T*) for TEG-enabled CFRP with 10 p-type CF-M40B thermocouples; (D) thermoelectric power at Δ*T* = 35, 55 and 75°C, with peak power of 0.87 μW at Δ*T* = 75 K when RLoad matches internal TEG resistance of 109.98 Ω. Reproduced with permission from [45]. © 2019 Elsevier.

CF-M40B outperforms CF-A38 in thermoelectric properties, including Seebeck coefficient, power factor and electrical conductivity. The TEG-enabled CFRP demonstrates effective thermal energy harvesting capabilities while maintaining acceptable mechanical performance, making it a promising material for integrated structural and energy-harvesting applications. Figure 3 investigates the thermoelectric and mechanical properties of TEG-enabled CFRP [45]. Figure 3A shows that the Seebeck coefficient (S) of CF-M40B is significantly higher than that of CF-A38, indicating superior thermoelectric performance. Both materials exhibit negative Seebeck coefficients, confirming n-type behaviour with electrons as the dominant charge carriers. Figure 3B highlights that the power factor (PF = *S*²*σ*) of CF-M40B is considerably higher than CF-A38, making it more suitable for TEG-enabled CFRP applications. Figure 3C demonstrates a linear increase in thermoelectric voltage with rising temperature difference (Δ*T*), aligning experimental data closely with simulation results and validating real-world thermal energy harvesting feasibility. Figure 3D shows that thermoelectric power output peaks at 0.87 μW at Δ*T* = 75°C when load resistance matches internal TEG resistance, confirming better performance under larger thermal differentials.

Optimized porosity yielded Seebeck coefficients of 62−72 μV K^−1^ and thermal conductivity reductions up to 30%. Short CF-epoxy composites demonstrated p-type thermoelectric behaviour, with Seebeck coefficients of 47 μV K^−1^. Increased CF content enhanced resistive heating and thermoelectric efficiency under mechanical strain [49]. Doping with Bi₂Te₃ significantly improved TEP, achieving up to 150 μW (m·K²)^−1^ via gradient mixing strategies [50]. Recent advances include polymer–carbon composites with 1D/2D fillers for enhanced thermoelectricity. These organic hybrid materials offer flexibility, lightweight properties and efficient charge transport—ideal for portable, structural and wearable thermoelectric systems [51]. These findings highlight the versatility of CFCs in thermoelectric energy harvesting and their adaptability for diverse applications, including wearable electronics, smart infrastructure and sustainable power generation platforms.

### 5.3. Piezo-electrochemical energy harvesting

The piezo-electrochemical (PEC) effect arises when mechanical stress induces electrochemical phenomena, such as ion migration or redox reactions, particularly in CFCs. These materials combine high mechanical strength and conductivity, enabling strain-induced charge separation and ion redistribution at fibre–matrix interfaces, producing electrical output analogous to the piezoelectric effect [52]. Lithium-ion intercalated CFCs (LICFCs) have shown superior PEC efficiency, with energy densities up to 10 times greater than traditional piezoelectric materials. Reported power densities reach 0.12 W cm^−2^ under strain, benefiting from high CF conductivity (>10 S cm^−1^) and aligned architectures that facilitate charge transport [52]. T800 fibres under tensile strain show state of charge (SOC)-dependent open-circuit potential shifts, with optimal PEC response at intermediate SOCs [53]. Longitudinal fibre expansion during lithiation and stress modelling support their integration into structural battery systems [54,55].

The PEC effect is especially advantageous at low frequencies, such as in wearable devices and EVs. CF-based PEC systems have powered humidity sensors (0.15 mW) and wireless sensor nodes (0.6 mW) operating for over two weeks without batteries [52,56]. LICFCs embedded in solid-state electrolytes respond to tensile and compressive loads, expanding applications across diverse environments [57]. Structural and electrochemical optimization is key. Modifications like conductive polymer coatings (e.g. polypyrrole (PPy), polyaniline (PANI)) and graphene integration improve ion transport and energy efficiency by 20−25% [58]. Hybrid systems combining PEC and triboelectric effects show up to 50% improved efficiency [53].

Stabilizing coatings extend PEC system lifespan by 40% [59], and CF bundles charged against lithium-metal counter electrodes harvest energy via reversible strain-induced voltage shifts, achieving 1 μW g^−1^ output [53].

**Figure 4.**
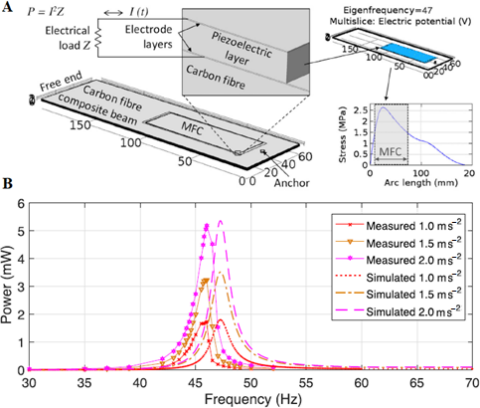
(A) Finite element model for PEC energy harvesting in MFC-integrated CFCs beam; and (B) frequency-dependent power output and resonance behaviour of the MFC transducer for PEC energy harvesting. Reproduced with permission from [60]. © 2019 Elsevier.

Figure 4 presents finite element modelling and analysis of PEC energy harvesting in macro fibre composite (MFC)-integrated CF structures. The finite element model (figure 4A) simulates the MFC transducer’s response, incorporating piezoelectric, CF and electrode layers to convert mechanical strain into electrical energy. Dynamic deformation generates alternating current, enabling power computation across an external load. Stress distribution and eigenfrequency (47 Hz) influence efficiency. Figure 4B compares experimental and simulated frequency responses, showing strong agreement (2.6% deviation) in resonance (47–48 Hz) and power output under varying accelerations. Results validate the model and highlight strain-induced charge separation for optimized energy harvesting [60].

## 6. Carbon fibre-based structural batteries: integrating energy storage and mechanical load-bearing

Multifunctional materials represent an emerging paradigm in materials science, enabling the simultaneous fulfiment of multiple engineering functions within a single material system. SBs are a prime example, integrating energy storage capabilities with mechanical load-bearing properties, thereby reducing overall system weight and enhancing efficiency in aerospace, automotive and wearable electronics applications. The SB concept is analogous to conventional fibre-reinforced composite laminates, where CFs serves as both high-performance mechanical reinforcements and electrochemically active components. These fibres are arranged in a layered architecture comprising a negative electrode, separator and positive electrode. On the negative electrode side, CFs facilitate the intercalation and insertion of Li-ions, functioning similarly to advanced Li-ion battery anode materials, such as graphite [61]. Their dual role as both structural reinforcements and active charge carriers enhances mechanical stability while maintaining electrochemical performance. The separator, typically composed of a thin glass fibre weave or vail, prevents electrical short circuits while maintaining ionic conductivity. On the positive electrode side, CFs are coated with an electrochemically active material, such as LiFePO₄, which ensures stable charge storage and discharge characteristics.

**Figure 5.**
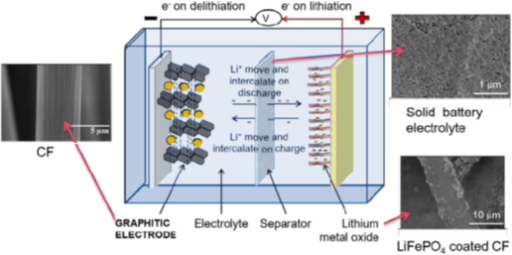
Principal concept for carbon fibre based structural batteries. Reproduced with permission from Zenkert *et al.* [62]. © ICCM.

To achieve both mechanical robustness and efficient ionic transport, the laminate stack is embedded within a structural battery electrolyte. This electrolyte must exhibit high ionic conductivity while also providing sufficient mechanical integrity to facilitate stress distribution under mechanical loads. Polymer-based or gel electrolytes are often employed to balance these requirements. Recent advancements in electrolyte design have focused on enhancing the interfacial compatibility between the fibre network and the electrolyte matrix to improve both electrochemical performance and mechanical durability [61]. Figure 5 illustrates the concept of CF-based SBs, which combine energy storage with mechanical load-bearing functionality. CFs act as both the negative electrode (graphitic anode) and structural reinforcement, while a lithium metal oxide cathode completes the electrochemical cell. During charging, Li^+^ move from the cathode and intercalate into the CFs, and during discharge, they de-intercalate and return to the cathode, generating electrical energy, with electrons (e⁻) flowing through an external circuit. A solid-state electrolyte and separator enable ion transport while ensuring electrical insulation. The inset images highlight key components: uncoated CFs (5 µm scale), LiFePO₄-coated CFs as the cathode (10 µm scale) and the solid electrolyte (1 µm scale), which enhances safety and mechanical integrity. This multifunctional design is critical for lightweight energy storage in aerospace, automotive and high-performance industries, offering both structural strength and energy storage while reducing system weight. By using the high strength and stiffness of CFs, SBs represent a transformative advancement in energy-efficient, load-bearing materials [62].

While CFs are renowned for their exceptional mechanical strength (tensile strength >3 GPa) and high electrical conductivity (>100 S cm^−1^), their low surface area of approximately 0.2 m² g^−1^ poses a significant limitation for energy storage applications, such as supercapacitors. Studies indicate that CFs needs a surface area of at least 100 m² g^−1^ to meet the energy storage requirements of structural supercapacitors. Experimental data reveal that CF-based supercapacitors with low surface areas achieve energy densities below 1 Wh kg^−1^, falling short of desired benchmarks. Although surface area enhancement through activation or hybridization with high-surface-area materials like graphene or CNTs can improve energy storage performance, these modifications often compromise mechanical properties [63]. To overcome this challenge, surface activation techniques have been explored. For instance, chemical activation has been shown to increase the surface area of CFs to over 200 m² g^−1^, significantly enhancing their charge storage capabilities and making them more suitable for structural supercapacitors. Notably, methods such as nitric acid treatment have achieved a 300% increase in surface area, reaching values of 180−220 m² g^−1^, which substantially improves their electrochemical performance [64]. While reference [65] highlights the effectiveness of such activation methods, further clarification is needed to fully understand the specific approach employed in this context.

The integration of GO with CFs has demonstrated significant enhancements in both electrochemical and mechanical properties. In one study, electrochemical oxidation was employed to modify CF surfaces, improving interfacial bonding with GO and epoxy matrices. The treated CF-GO composites exhibited a tensile strength increase of approximately 20%, rising from 3.5 GPa for untreated CFs to 4.2 GPa. Additionally, the interfacial shear strength improved from 40 MPa to 55 MPa, demonstrating enhanced load transfer efficiency within the composite structure. This modification not only strengthened the mechanical properties but also contributed to an increase in electrical conductivity from 1.5 × 10³ S m^−1^ to 2.2 × 10³ S m^−1^, making CF-GO composites highly suitable for multifunctional applications, including structural energy storage systems [66]. Furthermore, GO modification significantly influenced the electrochemical properties of CF-based electrodes. The hierarchical structure formed by the GO coating enhanced charge transfer kinetics, leading to an increase in specific capacitance from 80 F g^−1^ for untreated CFs to 150 F g^−1^ at a current density of 1 A g^−1^. The capacitance retention after 5000 charge–discharge cycles improved from 85% to 93%, indicating superior cycling stability. Additionally, the introduction of GO increased the composite’s surface area from 250 m² g^−1^ to 520 m² g^−1^, facilitating higher electrochemical activity. These improvements highlight the potential of CF-GO composites for applications requiring both mechanical durability and high electrochemical performance, such as structural supercapacitors and multifunctional energy storage devices [67].

SBs feature a composite design where CFs act as both structural elements and electrodes. The negative electrode is CF-based, while the positive uses CFs coated with active materials. A porous separator, like aramid or glass fibres, is embedded in an ion-conductive matrix to enable ion transport. Recent developments have shown that these separators can be optimized not only for ionic conductivity but also for structural performance. The separator must be as thin as possible to ensure that energy storage and power delivery are not compromised while maintaining the mechanical strength of the battery. Research into the use of advanced separator materials, such as aramid fibres, has suggested that they can provide both the necessary electrical insulation and mechanical reinforcement, contributing to the overall strength and stiffness of the composite [68]. A separator with a thickness of 30 μm was found to optimize performance, balancing both the mechanical and electrochemical properties of the battery. Despite the progress in modifying CFs and separators, the main challenge in developing CF-based SBs lies in balancing the mechanical and electrochemical properties. To maximize the efficiency of energy storage, the CFs must also function as current collectors, eliminating the need for traditional metallic current collectors. This results in weight savings and reduced material complexity, but it also necessitates precise optimization of the electrode material and ion-conductive matrix to ensure both high performance and structural integrity [69].

The key to realizing the full potential of CF-based SBs lies in continued research into surface modification techniques and the integration of advanced nanomaterials. By further enhancing the surface area of CF-GO or CFs-CNTs, and optimizing the design of ion-conductive matrices, the performance of these batteries can be significantly improved. Achieving this balance of high mechanical strength and efficient energy storage will be crucial for the development of next-generation multifunctional SBs [70]. In addition to ion intercalation, the integration of structural battery electrolytes (SBEs) with CFCs further enhances electrochemical optimization. SBEs, which serve as ionic conductors, provide a stable medium for ion migration while preventing issues such as dendrite formation and leakage that are often encountered with liquid electrolytes. By incorporating SBEs into the CFCs matrix, ions are more effectively mobilized during deformation, ensuring that the electrochemical processes can occur with minimal resistance, thus optimizing the energy conversion efficiency. The SBE plays a pivotal role in enabling the multifunctional nature of SBs, as it acts as both the electrochemical medium for ionic transport and the structural matrix for mechanical load-bearing. To fulfil these dual roles effectively, the SBE must maintain a delicate balance between ionic conductivity and mechanical strength, which often presents a significant challenge in its design. Early attempts to develop SBEs focused on homogeneous polymer thermosets incorporating ethylene oxide segments. These segments facilitated Li-ion coordination and transport, while the polymer network provided the necessary structural integrity [71]. Improving ionic conductivity often requires increasing the flexibility of the polymer electrolyte, which in turn reduces its mechanical rigidity. This trade-off creates a significant limitation in achieving optimal performance for SBEs, as both high ionic conductivity and mechanical strength are essential for their functionality. Studies have shown that while the use of plasticizing co-solvents can enhance ionic conductivity, they simultaneously compromise the structural integrity of the electrolyte. For instance, research demonstrated that incorporating plasticizing co-solvents improved ionic conductivity to values exceeding 10⁻³ S cm^−1^ at room temperature, but this came at the cost of reducing the elastic modulus by over 50% compared with unmodified polymer electrolytes [72]. These results emphasize the need for innovative material designs that can balance these competing properties to advance the performance of SBEs.

To address the limitations of mechanically weak solid-state batteries (SBEs), particularly in terms of their mechanical strength and ionic conductivity, researchers have investigated the use of nanocellulose as a reinforcement material. Nanocellulose, owing to its high specific surface area, biodegradability and mechanical properties, shows potential to enhance the overall multifunctional performance of SBEs. However, integrating nanocellulose into the battery matrix presents significant processing challenges, such as dispersion uniformity and interaction with the electrolyte, which complicates its effective incorporation into the system [73]. To overcome these issues, a more advanced technique, polymerization-induced phase separation (PIPS), has been employed. PIPS allows for the formation of a two-phase system at the nano-/micron scale, where the liquid phase serves as an ionic conductor to facilitate efficient ion transport, while the solid phase ensures structural stability and mechanical integrity. This two-phase system mirrors the approach used in structural supercapacitors, where the balance between electrical performance and mechanical strength is crucial, thereby offering a promising pathway for the development of high-performance, mechanically robust SBEs [74]. The process begins by preparing a homogeneous mixture containing a difunctional methacrylate monomer, conventional electrolyte solvents and lithium salts. Upon polymerization, this mixture undergoes phase separation, forming two distinct phases: a solid polymer phase and a liquid phase. These phases percolate at the nano-/micron scale, creating a bi-continuous network where the solid phase provides mechanical strength, while the liquid phase facilitates ion conduction. This phase-separated structure significantly enhances both the mechanical and ionic properties of the system, enabling effective load transfer and ion transport simultaneously. Research has demonstrated that this approach can achieve ionic conductivities exceeding 10⁻³ S cm^−1^ at room temperature, while maintaining an elastic modulus of several hundred MPa, making it highly suitable for structural battery applications. This methodology has shown great promise in the development of functional half-cells for batteries, offering an advanced approach to improving the performance of SBEs. Furthermore, the multifunctional performance of various SBEs has been modelled using finite element analysis, with topology optimization demonstrating substantial improvements in key properties, particularly the elastic modulus and ionic conductivity. These findings underscore the potential of computational design strategies in further enhancing the performance of SBEs [75].

To enhance the mechanical properties of SBEs and develop multifunctional morphing materials, CFs are commonly integrated using vacuum infusion techniques, resulting in unidirectional laminas with advanced electrochemical and mechanical properties. For instance, Ye *et al*. [76] use the dual functionality of CFs, which act as both structural reinforcements and conductive electrodes. The electrochemical performance of these composites is critical, as they must facilitate efficient ion transport while maintaining structural integrity. The study reports that optimized CF-reinforced SBEs achieve ionic conductivities of up to 10⁻³ S cm^−1^, which is essential for effective energy storage and discharge. Additionally, the integration of CFs ensures stable electrochemical performance, with minimal degradation in ionic conductivity and mechanical properties even after 50 charge/discharge cycles. This stability is crucial for long-term applications, particularly in morphing structures that require consistent performance under repeated mechanical and electrochemical stress. The combination of high ionic conductivity and mechanical strength makes these composites promising for industrial applications, where both energy storage and structural load-bearing capabilities are required [76].

The fabrication of a complete structural battery cell requires the integration of a positive electrode, which is typically applied to CFs using techniques such as autoclave infiltration, dip-coating or electrophoretic deposition (EPD). These methods generally involve the incorporation of lithium iron phosphate (LFP) with conductive additives, such as carbon black, and a polymeric binder like polyvinylidene fluoride (PVDF). However, when CFs were coated with LFP using these deposition techniques, the resulting values fell short of the theoretical capacity of LFP. This suggests limitations in the electrode material’s performance, probably owing to factors such as incomplete use of the active material or suboptimal electrode structure. It is important to note that these electrodes have not yet been evaluated in the context of a complete structural battery cell, which limits the understanding of their practical performance in composite battery configurations [77]. An alternative approach employed exfoliated graphene oxide (EGO) as a binder, which significantly enhanced the electrochemical performance of CFs. The EGO-coated CFs was integrated into a full-cell configuration, demonstrating an energy storage capacity of 155 mAh g^−1^ based on the total mass of the electrode. This performance was attributed to the improved electrical conductivity and ion diffusion facilitated by the EGO coating, which also contributed to a high Coulombic efficiency of 99.5 % over 100 charge/discharge cycles. Additionally, the EGO coating enabled a stable cycling performance with capacity retention of 92% after 100 cycles, showcasing its potential for long-term use. However, the testing was conducted with liquid electrolytes, meaning that while the electrochemical performance was promising, critical mechanical properties—such as energy density and load-bearing capability—were not evaluated. This highlights a significant gap in the research, as mechanical performance is essential for the multifunctional application of SBs, where both electrochemical and mechanical functionalities must be optimized simultaneously. The study by Sanchez *et al*. [78] underscores the potential of EGO as a binder for enhancing electrochemical performance but also emphasizes the need for further investigation into the mechanical properties of such systems to enable their use in structural battery applications [78]. Developments included a layer-by-layer assembly method, where LFP and nano-cellulose fibrils (CNF) were used as binders. These fibres were carbonized to form an electrically conductive carbon network, which enabled higher LFP loading (up to 70 wt%) and a faster production process. This technique demonstrated significant potential for improving both the capacity and manufacturing speed for structural positive electrodes. Specifically, the resulting electrodes achieved a specific capacity of 160 mAh g^−1^ at 0.1°C, with excellent rate capability, retaining 85% of the initial capacity at 1°C. The carbonized CNF network also provided enhanced mechanical stability, with a tensile strength of 120 MPa, making it suitable for structural applications. This approach highlights the potential for scalable and efficient production of multifunctional composite materials that combine energy storage and structural integrity [79].

The development of CF-based structural battery composites, which aim to combine energy storage capabilities with mechanical performance, has seen significant progress but remains challenged by the inherent trade-off between energy storage and mechanical strength. For instance, traditional energy storage systems like Li-ion batteries achieve energy densities of up to 200 Wh kg^−1^ or more, coupled with an elastic modulus in the range of 10−20 GPa. By contrast, CF-based composites exhibit significantly lower energy densities owing to limitations in charge-storage materials, such as reduced ion conductivity and lower specific capacity. Furthermore, efforts to enhance energy density often result in a decline in mechanical properties, reducing the material’s ability to bear heavy loads or withstand stress. For example, conventional CFCs, designed primarily for structural strength, can achieve elastic moduli exceeding 100 GPa, underscoring the substantial compromise involved when integrating energy storage functionality into these materials [80]. In a more recent study, a structural battery cell was developed that achieved an energy density of 1.4 Wh kg^−1^ and a significantly improved elastic modulus of 7 GPa. This marked a notable advancement compared with earlier CF-based structural battery composites, which typically exhibit much lower elastic moduli, often in the range of 0.2−0.5 GPa. However, despite this improvement in mechanical properties, the energy density of 1.4 Wh kg^−1^ remains significantly lower than that of traditional energy storage systems, such as Li-ion batteries, which can achieve energy densities of 150−250 Wh kg^−1^. This result highlights the persistent challenge of balancing energy storage capacity with mechanical performance in multifunctional structural battery composites [81].

The study highlights that while mechanical strength can be significantly improved—approaching the elastic moduli of conventional structural materials like metals (e.g. steel with a modulus of approx. 210 GPa)—energy density remains limited owing to inherent constraints in energy storage materials. Enhancing mechanical properties often involves incorporating high-performance structural components, which can reduce the volume available for energy storage. As a result, achieving both high energy density and superior mechanical strength remains challenging. Recent strategies aim to improve energy storage materials, such as using high-capacity electrode materials or developing hybrid composites, which could offer a better balance between these two critical performance metrics. Despite advancements, further innovation is needed to bridge the gap between energy density and mechanical strength, with future efforts probably focusing on optimizing the design and material composition of multifunctional composites [65]. In comparison with current commercial LFP-based batteries, which boast energy densities of 130−150 Wh kg^−1^ and longitudinal moduli ranging from 100 to 150 GPa in uni-directional CF composites, the performance of structural battery concepts remains significantly behind. These commercial batteries, designed primarily for high energy storage and mechanical stability, far exceed the multifunctional performance achieved by CF-based SBs to date. For instance, a typical LFP-based battery can offer much higher energy densities and superior mechanical properties owing to the mature technologies used in electrode materials and structural composites. By contrast, structural battery designs that integrate energy storage and mechanical strength into a single material still face the challenge of balancing these two functions effectively, as seen in the reported results [82]. While these results are promising, they still fall short of the performance levels seen in purely CF-based SBs, which have yet to achieve substantial multifunctional performance. This underscores the difficulty of designing a fully CF-based structural battery that can compete with commercial energy storage systems in terms of both energy density and mechanical strength.

A thermodynamically consistent modelling framework, initially developed for conventional batteries, has been adapted to simulate the electro-chemo-mechanical properties of laminated structural battery negative half-cells [83–85]. A galvanostatic charge–discharge model for lithium-polymer-insertion cells was developed, offering critical insights into ion transport, electrode kinetics and concentration-dependent diffusion coefficients. The study revealed that discharge capacity is significantly influenced by the electrolyte’s ionic conductivity and the solid-phase diffusion of Li^+^, which directly impact overall electrochemical performance. Furthermore, the model highlighted the importance of optimizing electrode–electrolyte interfaces to minimize polarization losses and enhance charge transfer efficiency. It also emphasized the role of concentration gradients in determining the rate capabilities of the cell, particularly under high-current conditions. Additionally, the study underscored the need for advanced electrolyte formulations to improve ionic mobility and reduce interfacial resistance, which are critical for achieving higher energy densities and longer cycle life in lithium-based batteries [83].

A porous-electrode theory was developed to incorporate electrolyte transport, electrochemical reaction rates and charge conservation, providing a foundational framework for battery modelling. This approach quantified the influence of electrode porosity on charge–discharge behaviour and illustrated how reaction distribution varies across the electrode thickness, ultimately affecting energy density and power output [84]. Carlstedt *et al.* extended these principles to SBs, demonstrating that mechanical loading significantly affects electrochemical performance [85]. Their model revealed that an applied mechanical strain of 0.3% could lead to a 15% reduction in ionic conductivity, while internal stress accumulation alters charge transfer kinetics. Additionally, the study predicted an energy storage capacity of approximately 25−35 Wh kg^−1^ for structural battery composites, which remains lower than conventional Li-ion batteries but offers structural integration benefits. The longitudinal modulus of a unidirectional CF composite used in SBs was estimated to be around 120 GPa, providing sufficient stiffness for load-bearing applications. However, increasing energy density often compromises mechanical integrity, requiring careful optimization of material interfaces and hybrid reinforcements. These findings highlight the strong coupling between mechanical and electrochemical processes, emphasizing the challenge of maintaining both energy storage efficiency and mechanical integrity in structural battery designs. Furthermore, studies have investigated the impact of migration, stress-assisted diffusion and convection within the electrolyte on the overall electrochemical and mechanical behaviour of SBs. These factors are critical, as they influence ion transport efficiency, stress distribution and the overall functionality of the composite. Notably, findings have revealed that convective effects have a minimal impact on multifunctional performance under relevant mechanical loads, suggesting that mechanical stresses and electrochemical reactions are more dominant in determining battery performance. For instance, numerical simulations demonstrated that stress-assisted diffusion enhances ion transport by up to 15% under high mechanical loads, while convection contributes less than 5% to the overall ion flux [86].

Additionally, heat generation during galvanostatic cycling, particularly at the fibre/electrolyte interface, has been experimentally validated. Discontinuities at this interface can lead to localized heating, which, if not properly managed, may degrade both electrochemical and mechanical performance. Experimental data showed that temperature spikes of up to 10°C can occur at the interface during high-rate cycling, potentially accelerating degradation mechanisms [87]. The expansion of CFs in the negative electrode owing to lithium insertion induces residual strains that affect the material’s mechanical properties. These strains, which can reach up to 0.5% in some cases, have been confirmed through both numerical simulations and experimental studies, highlighting their importance for improving the long-term stability and performance of SBs. For example, residual strains were found to reduce the tensile strength of the composite by approximately 8% after 100 charge–discharge cycles [87]. By integrating these multi-physics phenomena, such models provide a more comprehensive understanding of the material’s behaviour, paving the way for future innovations and optimizations in structural battery technology. For instance, incorporating stress-assisted diffusion and thermal management strategies into the design process has been shown to improve energy density by up to 20% while maintaining structural integrity [86].

The potential mass savings from SBs are significant, particularly in applications where reducing weight is crucial for performance and energy efficiency. Theoretical predictions suggest that replacing a steel roof in a battery electric vehicle (BEV) with a structural battery could save up to 40–50% of the roof’s mass, leading to substantial reductions in overall vehicle weight. By contrast, replacing a CF hull structure in an electric ferry with a structural battery would only save 10–15% of the mass, highlighting the varying impact of SBs across different types of applications [88]. Similarly, in consumer electronics such as laptops, SBs could potentially eliminate the need for a separate, conventional battery, offering both energy storage and structural support within a single component. These mass savings, in turn, could lead to 5–10% lower energy consumption, thus reducing the environmental impact of EVs. In the case of BEVs, integrating structural battery composites could result in up to 15% more energy-efficient vehicles with extended driving ranges [89], addressing key challenges in the EV industry. The performance analysis framework for SB composites evaluates the balance between energy storage and mechanical strength required for effective vehicle design. This framework quantifies how SBs, which serve as both energy storage and structural components, compare with traditional battery systems by assessing their efficiency and performance in EVs. For example, SBs have been shown to achieve energy densities of up to 200 Wh kg^−1^ while maintaining tensile strengths exceeding 500 MPa, making them competitive with conventional Li-ion batteries in terms of energy storage and superior in terms of mechanical performance. By integrating SBs, significant weight reduction and space optimization are achieved, improving overall energy efficiency and extending the driving range of BEVs. The framework addresses the trade-off between the mechanical and electrochemical properties of SBs, guiding the selection of materials and design for optimal energy density and mechanical support, which are essential for maximizing both performance and safety in EV applications.

Efforts to develop CF-based positive electrodes are advancing through innovative coating strategies, particularly those using active materials like LiFePO4. These coatings enhance the electrochemical performance of CFs, thereby enabling their dual functionality as both structural components and energy storage elements. A key advancement in this area is the EPD of LiFePO4/GO composites on CFs, which has demonstrated improved capacity and energy efficiency while maintaining structural integrity. Similarly, powder impregnation methods, where CFs are impregnated with lithium iron phosphate (LiFePO4) powders, have shown promise for achieving uniform coating distribution, albeit with challenges related to scalability and process optimization [65,78–81]. The development of robust adhesion between the coating and the CF surface remains a critical challenge. Factors such as surface chemistry, roughness and the nature of the binder significantly influence the interfacial stability and electrical conductivity of the coated electrodes [79]. Moreover, transitioning from batch to continuous coating processes is essential for large-scale production. Continuous methods, such as EPD or roll-to-roll techniques, are being actively explored to address scalability while ensuring consistent coating quality [78].

Recent advancements in manufacturing techniques, such as screen printing, have shown significant promise in improving the functionality and efficiency of CF-based structural batteries. Johannisson *et al*. [90] demonstrated a screen-printing method to fabricate lightweight metal current collectors directly onto CF electrodes. This approach reduces the overall mass of the system while maintaining high electrical conductivity and compatibility with the mechanical properties of the CFs. By eliminating the need for additional adhesive layers or bulky components, screen printing streamlines the production process, making it more efficient and scalable. Furthermore, the integration of printed current collectors enhances the system’s structural integrity, contributing to the development of multifunctional batteries that combine energy storage with load-bearing capabilities. These innovations highlight the importance of exploring advanced manufacturing techniques to optimize both the electrochemical performance and structural efficiency of CF-based systems. Screen printing, as a versatile and scalable method, represents a pivotal step toward achieving high-performance, lightweight SBs for next-generation applications [90].

The development of advanced electrolyte systems has further highlighted the critical role of encapsulation in ensuring the performance and longevity of SBEs. One notable advancement is the use of bicontinuous electrolytes formed via thermally initiated polymerization, which provide enhanced compatibility with structural laminates. These electrolytes possess a dual-phase architecture, allowing for efficient ionic conductivity while maintaining the mechanical integrity of the composite structure. However, such systems require sophisticated encapsulation strategies that not only protect the electrolyte from external environmental factors, such as moisture and contaminants, but also preserve its unique properties, such as ionic transport pathways and mechanical cohesion, over extended periods [91]. Unidirectional CF lamina electrodes represent another significant innovation in SBEs. These electrodes integrate structural and electrochemical functionalities, making them a cornerstone of multifunctional energy storage systems. However, the performance of these electrodes is highly dependent on effective encapsulation, which ensures stability and prevents degradation caused by environmental exposure. Encapsulation is vital for maintaining the efficiency of the electrodes by acting as a barrier against moisture ingress and facilitating mechanical load transfer within the composite system [92]. In terms of environmental impact, SBEs represent a transformative solution to reduce the carbon footprint in applications like BEVs. Lifecycle assessments reveal that SBs not only integrate energy storage with load-bearing functionalities but also significantly reduce CO₂-equivalent emissions. This dual-purpose capability reduces material usage and overall system weight, contributing positively to sustainability in green technology [93].

Current research focuses on enhancing manufacturing techniques such as resin vacuum infusion and coextrusion printing, which have shown significant potential in producing high-performance structural electrodes. Resin vacuum infusion involves placing dry CF reinforcements into a mould, followed by infusing the fibres with a liquid resin under vacuum. This technique ensures uniform resin distribution, reduces void content and enhances the mechanical integrity of the composite. It allows seamless integration of CFs with active materials, creating components that are both mechanically robust and electrochemically efficient. Scalable, cost-effective and compatible with various matrix and electrode materials, resin vacuum infusion is ideal for structural battery electrodes [94]. Coextrusion printing simultaneously deposits continuous CFs and active material slurries to create three-dimensional structured composites. This additive manufacturing approach enables precise control over fibre and material placement, ensuring optimal alignment and distribution. It allows the creation of complex geometries, maximizing electrode surface area and energy density. With high throughput and customization, coextrusion printing holds promise for scalable production of multifunctional electrodes [95]. Both techniques combine mechanical and electrochemical functionalities, creating lightweight and efficient material systems suitable for SBs. Further development and optimization could lead to transformative improvements in the design and manufacture of energy storage devices.

The evolution of CFCs for energy-related applications has been significantly accelerated by the incorporation of nanoscale additives. While conventional CFCs offer excellent tensile strength, low density and high corrosion resistance, their relatively low surface area and modest electrochemical activity impose limitations on their applicability in high-performance energy storage and harvesting devices. These traditional systems typically demonstrate moderate energy and power densities, and their specific capacitance remains below the thresholds required for advanced multifunctional systems. By contrast, hybridization with nanomaterials has emerged as a transformative approach that re-engineers the ion transport pathways and charge storage mechanisms within the CFC matrix. This includes the integration of carbon-based nanomaterials (e.g. graphene, CNTs), metal oxides (e.g. MnO₂, TiO₂), and conductive polymers (e.g. PANI), which collectively enhance the electrochemical interface, provide abundant active sites and improve overall device performance.

The incorporation of nanomaterials not only increases surface reactivity but also enables the formation of percolated conductive networks, which reduce charge transfer resistance and accelerate redox kinetics. As a result, nanomaterial-enhanced CFCs exhibit superior performance across multiple metrics, including specific capacitance, electrical conductivity, energy density and power density. For instance, the energy density can exceed 150 Wh kg^−1^ in systems using MnO₂ or graphene-based pseudocapacitive materials—far beyond the 10−50 Wh kg^−1^ range typical for unmodified CFCs. Specific capacitance also experiences an order-of-magnitude improvement, from 50−200 F g^−1^ to more than 1000 F g^−1^ in optimized nanostructured configurations. Notably, these enhancements are achieved without a detrimental impact on mechanical performance; in many cases, reinforcement with nanomaterials even improves tensile strength and fatigue resistance owing to better load transfer at the fibre–matrix interface.

Table 3 summarizes the comparative performance characteristics of conventional and nanomaterial-enhanced CFCs, with a focus on key parameters relevant to electrochemical energy systems. The performance advantages conferred by nanostructured modifications are evident in all major categories, including energy and power density, conductivity and long-term cycling stability. The table also reflects the ability of these hybrid composites to maintain or enhance mechanical strength while offering new functionality. These findings reinforce the conclusion that nanomaterials are not auxiliary additives but are essential design elements for the realization of next-generation structural energy storage systems. As the field advances, scalable processing methods—such as electrophoretic deposition, roll-to-roll fabrication and three-dimensional hybrid printing—are expected to bridge the gap between laboratory demonstrations and commercial deployment.

**Table 3.** Comparative performance metrics of conventional versus nanomaterial-enhanced CFCs in structural electrochemical applications.

property	conventional CFCs	nanomaterial-enhanced CFCs	performance improvement mechanism	references
energy density (Wh kg^−1^)	10–50	50−150+ (with MnO₂, graphene, GO)	increased surface area and pseudocapacitive contribution	[63,75,80]
electrical conductivity (S m^−1^)	10³–10⁵	10⁴−10⁶ (via CNTs, MXenes)	enhanced charge mobility through conductive nanonetworks	[67,68,77]
cycling stability (cycles)	1000–5000	5000−20 000 (with Co₃O₄, PANI, etc.)	improved ion diffusion and structural resilience	[64,69,74]
specific capacitance (F g^−1^)	50–200	200−1000+ (hierarchical nanostructures)	larger number of active sites and redox synergy	[66,70,73]
power density (W kg^−1^)	500–2000	2000–10 000	faster charge/discharge enabled by lower internal resistance	[63,72,74]
tensile strength (MPa)	3500–7000	4000−8000 (reinforced with CNTs/GO)	retention or enhancement of mechanical strength	[67,71]
interfacial bonding strength (MPa)	30–45	50−80 (GO, functional CNTs, plasma-treated)	improved matrix–fibre adhesion and load transfer	[68,69]
electrochemical stability window (V)	1.5–2.5	2.5−3.8 (graphene, MXenes, doped CFs)	broadened voltage range for stable high-capacity operation	[72,75]

## 7. Transforming shape-morphing and energy storage with carbon fibre composites actuators

CF-based materials' inherent anisotropy, combined with electrochemical and thermal responsiveness, enable controlled deformation under external stimuli. By using the piezoelectric electrochemical transduction (PECT) effect, CFs can undergo reversible expansion and contraction when subjected to electrical or chemical stimuli, enabling dynamic shape transformations. This expansion is driven by ion intercalation within the fibre structure, as seen in SBs and electrochemical actuators, allowing for precise deformation controlled by applied voltage. This mechanism eliminates the need for bulky mechanical components, offering a more efficient and adaptable solution for shape-morphing applications. Additionally, integrating polymeric or nanocomposite coatings on the fibres further enhances actuation performance, improving flexibility and ionic transport. While piezoelectric materials also enable mechanical deformation in response to electrical signals, they are often non-structural and must be added as layers or patches, which increases system weight and complexity. These materials are commonly used in high-frequency actuation systems but require high operating voltages, complicating integration with other electronic systems. Furthermore, their performance is constrained by intrinsic mechanical properties and the need for precise voltage control, which may not always be feasible in dynamic environments [96,97].

Solid-state actuators, including piezoelectric materials, shape-memory alloys (SMAs) and pseudocapacitive materials, offer promising alternatives for shape-morphing applications, providing significant advantages over traditional static designs. These materials enable deformation with reduced mass and complexity compared with conventional actuation systems. However, challenges such as limited strain output, response time and integration with load-bearing structures remain key obstacles to their widespread adoption in structural applications. Researchers continue to explore innovative strategies to optimize these materials for enhanced efficiency, adaptability and multifunctionality. The SMAs, another class of actuation materials, work by undergoing a reversible phase transformation when exposed to a specific temperature or pressure change. While SMAs are capable of producing significant actuation forces, they require external stimuli, such as heating or cooling, to trigger the transformation. This makes them less suitable for applications requiring continuous or rapid actuation in varying environmental conditions. Additionally, the phase transition in SMAs is often relatively slow, and the material’s response is time-dependent, which can limit their effectiveness in certain dynamic systems [96].

Pseudocapacitive materials, which are integral to electrochemical actuation, offer a promising alternative to traditional mechanical actuators owing to their unique ability to undergo electrochemical reactions, such as ion insertion and extraction that induce volumetric changes. These materials, including transition metal oxides and conducting polymers, are characterized by their high specific capacitance and fast electrochemical response, enabling high actuation forces even at low voltages. Nanostructured polymer electrolytes were investigated for their potential in enabling fast, low-voltage electroactive actuators. The incorporation of these materials resulted in improved performance, particularly in terms of response speed and energy efficiency. The study found that nanostructured electrolytes enhance ion transport properties, which play a critical role in increasing actuation force and speed while operating at lower voltages [97]. On the other hand, carbon nanotube actuators, which exhibit significant pseudocapacitive behaviour, were demonstrated to achieve high actuation forces, making them highly suitable for applications in flexible electronics and robotics. These actuators operate through electrochemical charge storage mechanisms at the carbon nanotube interface, enabling a rapid response to electrical input. However, the study also identified limitations related to energy consumption, as continuous power input is necessary to sustain actuation, particularly in configurations with high energy demands [98].

Further studies, such as the work by Gu *et al.*, also investigated nanofibre sheet actuators based on vanadium pentoxide (V_2_O_5_), a well-known transition metal oxide. These V_2_O_5_-based nanofibre actuators exhibited substantial pseudocapacitive actuation, with the added benefit of high energy density and faster response times compared with traditional materials [99]. Their study underscored the importance of optimizing material properties, such as ion diffusion rates, to improve actuator efficiency. V_2_O_5_ was found to perform particularly well in fast, low-voltage applications, though it still faced challenges related to long-term stability and energy consumption. Carbon nanotube and graphene-based bioinspired electrochemical actuators were explored for their high efficiency and versatility, with a focus on using the conductive properties of carbon-based materials. These materials are particularly suitable for applications requiring low power input and high mechanical performance. The study concluded that such materials could function as effective electrochemical actuators, capable of delivering efficient and rapid actuation while maintaining a relatively low energy footprint. However, it was noted that energy consumption remains a challenge, especially in systems requiring continuous deformation, which limits their practical use in energy-efficient applications [100].

Overall, these studies demonstrate that pseudocapacitive actuators, although promising in terms of high actuation forces and low-voltage operation, still face significant challenges in terms of energy consumption, long-term stability and reliance on liquid electrolytes. The ongoing development of solid-state electrolytes and optimization of materials will be crucial to overcoming these limitations and expanding the practical application of pseudocapacitive actuators in diverse fields. In response to these challenges, a new class of solid-state actuators based on volume changes caused by ion-insertion has gained attention as a potential solution for overcoming many of the limitations associated with traditional actuation mechanisms. These materials, which undergo significant volume expansions because of the intercalation of ions, offer the advantage of providing high actuation forces at low voltages without the need for a continuous external trigger. One of the most compelling benefits of ion-insertion-based actuators is their potential for ‘zero-power hold’, meaning that once the material has undergone deformation, it can maintain its new shape without requiring any further energy input. This property could significantly reduce the energy consumption of morphing structures, making them more efficient and sustainable for long-term use [101].

Despite these promising features, the use of ion-insertion materials for shape-morphing applications has traditionally been limited by the reliance on liquid electrolytes to facilitate ion transport. This reliance has constrained the material’s ability to function effectively in structural applications, where stability and mechanical strength are paramount. Liquid electrolytes can also introduce additional complexity and pose challenges related to leakage, temperature sensitivity and long-term reliability. However, recent advancements in solid-state electrolytes and intercalation compounds have begun to address these issues. By using solid-state ion conductors, researchers have successfully developed materials capable of undergoing ion-insertion and achieving substantial volume changes without the need for liquid electrolytes. These solid-state actuators offer the possibility of achieving high performance with minimal parasitic mass, making them ideal candidates for use in lightweight, multifunctional structures. One of the key innovations in this area has been the development of solid-state intercalation compounds, such as Li-ion insertion materials, that enable effective actuation without sacrificing structural integrity. Solid-state materials have been shown to exhibit large-volume changes, making them highly effective for actuation in systems where energy efficiency, durability and scalability are critical. By using ion-insertion to achieve reversible volume expansion and contraction, these materials enable shape-morphing without the complexity and high power demands of traditional actuation systems. The capability to achieve actuation at low voltages further reduces power consumption, which is particularly beneficial in applications such as aerospace, where energy efficiency and weight reduction are essential [102].

In addition to their actuation capabilities, ion-insertion-based materials offer the potential for multifunctional structures. For instance, carbon-based materials such as carbon nanotubes, graphene and CFs can be engineered to function as both structural components and actuation materials. By combining the mechanical strength of these materials with their ability to undergo volume changes owing to ion-insertion, researchers have begun to explore the integration of shape-morphing capabilities into lightweight composite materials. This has the potential to revolutionize the design of adaptive structures in areas such as wind-turbine blades, aerospace components and even soft robotics. In such systems, the same material could serve as both a load-bearing structural component and a responsive actuator, reducing the need for additional components and improving overall system efficiency. Despite the progress in developing ion-insertion-based actuation materials, challenges remain in terms of optimizing their performance, reliability and scalability. Much work is still needed to improve the materials' long-term stability, ion conductivity and mechanical properties to ensure that they can meet the demands of real-world applications. Additionally, developing cost-effective manufacturing processes for these materials will be essential to enable their widespread adoption in commercial applications.

The electrochemical performance of CFs with ion insertion, particularly with lithium, sodium and potassium ions, plays a crucial role in their application to multifunctional materials. This ion-insertion-induced volume expansion, which is vital for actuation and shape-morphing, is supported by the electrochemical behaviour of CFs in various energy storage systems. For potassium-ion insertion, the polyacrylonitrile-based CFs studied exhibited a specific capacity of 230 mAh g^−1^, reflecting their ability to store a significant amount of energy. The CFs also demonstrated excellent cycling stability, with a Coulombic efficiency of around 99% over 100 cycles. This high efficiency indicates that most of the inserted potassium ions are reversibly extracted, suggesting that CFs are well-suited for applications in energy storage and also for morphing devices, where the structural deformation could be used for actuation purposes. The potassium-ion insertion not only facilitates energy storage but also enables volume expansion and contraction, crucial for morphing and strain-sensing applications, making them suitable for low-voltage actuation devices [103].

In the case of Li-ion batteries, modified CFs used as structural anodes showed promising electrochemical performance with a specific capacity of 350 mAh g^−1^. These fibres were capable of maintaining stable performance over 100 cycles, achieving a Coulombic efficiency near 99%. This suggests that the CFs are highly efficient in terms of energy storage, and their electrochemical properties allow them to undergo reversible volume changes. These characteristics are essential for applications where both energy storage and actuation are needed. The high reversible capacity, coupled with minimal energy loss over cycles, makes CFs ideal for applications requiring continuous deformation and expansion, such as in morphing systems [104]. While Li-ion insertion is a widely studied system, there has been increasing interest in using Na and K ions for actuation and energy storage applications. Sodium and potassium have larger ionic radii compared with lithium, which might result in different insertion behaviour and volume expansion. Despite the larger size of Na and K ions, studies show that they induce smaller expansions in CFs compared with Li^+^s for the same amount of charge inserted [18]. This difference is attributed to the unique ion-insertion mechanisms that Na and K follow in the CF microstructure.

Na-ion insertion, for example, is characterized by a bilinear strain versus capacity relationship, which suggests that Na ions first insert into more ordered domains of the CF structure before occupying micropores at lower potentials. This contrasts with Li-ion insertion, which tends to result in more uniform ion distribution across the CF structure. The distinct ion insertion mechanisms for Na are further evidenced by the staging observed in the charge/discharge curves during Na-ion cycling in CFs [17]. Understanding these mechanisms is crucial for optimizing the actuation properties of CFs when using different ions. When investigating Na-ion batteries, CFs produced through forcespinning exhibited a reversible capacity of 150 mAh g^−1^ at a low current density of 0.1 A g^−1^. This capacity is significant for Na-ion batteries, which typically have lower energy densities compared with Li-ion systems. The CFs also demonstrated excellent cycling stability, retaining high capacity over 500 cycles. This resilience in cycling performance indicates that CFs can endure the physical stresses resulting from volume changes during Na-ion insertion without significant degradation. Such fibres are suitable for flexible and morphing systems, where mechanical deformation is essential, and the ability to maintain structural integrity during repeated ion insertion cycles is crucial [105].

Additionally, Na-ion capacitors made from CF cloth demonstrated a high specific capacitance of 150 F g^−1^ at 1 A g^−1^, which is indicative of their ability to store significant amounts of charge while exhibiting excellent rate capability. This suggests that CFs can be used not only for energy storage but also for applications that require rapid charge/discharge cycles. The performance of these capacitors aligns with the requirements of morphing materials, where fast responsiveness to electrical inputs is necessary for deformation or actuation. The ability to maintain this capacitance over long periods makes these CF-based capacitors ideal for flexible energy storage and morphing applications [106]. CF modifications for stable sodium metal anodes, which focus on optimizing interfacial chemistry and structural engineering, achieved a reversible capacity of 400 mAh g^−1^ at 0.2 A g^−1^. This performance demonstrates that the CFs can provide high energy storage while undergoing repeated volume changes without losing structural stability. Such properties are essential for both stable energy storage and actuation, as the fibres must endure significant expansion and contraction without failure. This ability to undergo controlled expansion is key to using CFs in applications where mechanical actuation is required [107].

K-ion insertion has also been explored, though the expansion is typically smaller than that seen for lithium. K-ions, like sodium, tend to show a different insertion mechanism in CFs, which requires further investigation to fully understand how they interact with the CF structure. This ongoing research into Na- and K-ion insertion suggests that they might be viable alternatives to lithium, especially for applications where cost, availability and environmental impact are key considerations. K-ion batteries using CF@CNT composites exhibited a specific capacity of 150 mAh g^−1^ at a current density of 0.1 A g^−1^. These composites showed good cycle stability and high rate capability, further confirming that CFs, particularly when enhanced with CNTs, can be used for both energy storage and actuation. The combination of CFs and CNTs improves the electrical conductivity and mechanical properties of the fibres, which is crucial for the durability and responsiveness needed in morphing materials [108]. The ability to control the expansion and contraction of CFs through ion-insertion opens up exciting possibilities for the development of multifunctional composite materials. These materials can combine energy storage capabilities with structural actuation, which is highly advantageous in applications such as morphing structures, lightweight actuators, strain sensors and even SBs. In these composites, the ion-insertion process not only provides the energy storage function but also acts as the driving mechanism for shape-morphing behaviours. For instance, ion-insertion in CFs can be employed in SBs, where CFs simultaneously acts as a load-bearing material and an energy storage medium. This dual functionality reduces the need for separate actuators or additional energy storage systems, resulting in more efficient, lightweight materials that serve multiple purposes. Additionally, the actuation generated by ion-insertion in CFs can be exploited for strain-sensing applications. As the CFs expand or contract in response to external forces, this change in volume can be used to monitor stress and strain within a structure, offering a means to assess the mechanical integrity of materials in real-time [109].

CFs undergo volume expansion when lithiated, which can be exploited for structural actuation purposes. This expansion occurs both in the longitudinal and radial directions, though the radial expansion is substantially larger. Despite this, the radial expansion is less relevant for structural actuation owing to the optimization of CFs’ strength and stiffness in the longitudinal direction. Consequently, the longitudinal expansion remains the primary focus for actuation applications. During Li-ion insertion, CFs exhibit small but significant longitudinal expansion, which is ideal for their use as linear actuators. The expansion in the longitudinal direction, though relatively minor, can generate large forces owing to the inherent stiffness of CFs. For instance, a 0.5% irreversible expansion results in a stress of approximately 1500 MPa [54]. This highlights CFs' potential as actuators capable of producing substantial forces with low electrical input. The energy density of these actuators is also notable. Assuming a CF with a modulus of 300 GPa and an irreversible expansion of 0.5% when inserted with Li^+^, the energy density achieved is 3750 kJ m^−3^. With a density of around 1750 kg m^−3^, these results in a specific energy density exceeding 2000 J kg^−1^, far are surpassing the energy density of conventional piezoelectric materials such as lead–zirconium–titanate (PZT) ceramics, which have a specific energy density of only 14 J kg^−1^ [75]. The volume expansion induced by Li-ion insertion, though relatively small, demonstrates the high force potential of CFs in actuation. The increase in stress, such as 1500 MPa owing to a 0.5% expansion, suggests that CFs can generate significant forces even with modest ion-insertion expansion. This characteristic is advantageous for creating actuators that operate at low voltages with inherent zero-power hold, making CFs suitable for applications requiring high mechanical performance and energy efficiency.

The integration of SBEs with CFs for developing shape-morphing laminates is fundamentally governed by electrochemical processes involving ion insertion and extraction. Electrochemical measurements have demonstrated that the insertion of Li^+^s into CFs induces volumetric expansion owing to intercalation, whereas ion extraction results in contraction. This bidirectional strain response generates differential expansion across the laminate structure, leading to controlled bending behaviour. The extent of deformation is directly correlated with the applied potential, ion concentration and cycling parameters, highlighting the tunability of electrochemical actuation. Galvanostatic cycling tests have confirmed that CF-based laminates exhibit stable charge–discharge profiles, with minimal capacity fading over multiple electrochemical cycles, ensuring reliable long-term actuation performance. Further electrochemical analysis has revealed that the bending deformation can be actively reversed by applying an alternating potential, allowing for programmable and cyclic shape morphing. Cyclic voltammetry (CV) studies indicate distinct redox peaks associated with the ion insertion/extraction process, confirming the reversible nature of electrochemical actuation. Electrochemical impedance spectroscopy (EIS) measurements further suggest that the integration of SBEs enhances ion transport kinetics, reducing interfacial resistance and improving overall electrochemical responsiveness. These findings underscore the multifunctionality of CF-based laminates, making them highly suitable for applications requiring dynamic reconfiguration [110].

The use of CFs for shape-morphing offers significant advantages, particularly in applications requiring large deformations and high forces with low-voltage input. Electrochemical investigations have demonstrated that CF-based structural battery laminates exhibit controlled deformation owing to ion insertion-induced expansion. In a recent study, CF laminates exhibited bending curvatures of 3.5 m⁻¹ when a charge capacity of 160 mAh g^−1^ was achieved under an applied voltage of 2.5 V. This bending behaviour is directly proportional to the charge capacity and applied voltage. Additionally, the materials were able to maintain their deformed shape after several cycles without requiring continuous power input, demonstrating a zero-power hold. After 100 charge–discharge cycles, the laminate showed minimal degradation, with a capacity retention of approximately 95%, confirming their potential for energy-efficient, long-term applications. This characteristic is particularly valuable in systems such as fluid control surfaces for aircraft wings or wind turbine blades, where precise and reliable shape adjustments are needed to optimize performance [111]. The versatility of CFs is further enhanced by their ability to alter fibre angles within composite layers, enabling complex morphing behaviours such as simultaneous bending and twisting. Analytical and numerical simulations have shown that varying the fibre orientations in CF laminates can influence the deformation modes significantly, allowing for tailored shape-morphing responses. Specifically, changing the fibre angle by 15−30° led to different strain distributions, enabling more complex morphing behaviours. Electrochemical strain analysis revealed that the insertion of ions results in localized stresses, which were optimized in the simulations to avoid mechanical degradation over extended cycles. Furthermore, EIS measurements demonstrated a low charge transfer resistance (approximately 40 Ω), ensuring efficient ion transport and rapid actuation. The integration of these findings into design and optimization processes ensures that CF-based SBs can withstand the mechanical and electrochemical stresses encountered during operation, making them highly suitable for dynamic, adaptive systems, such as morphing aircraft and flow control mechanisms [112].

## 8. Role of nanomaterials in carbon fibre composites for electrochemical applications

Nanomaterials, particularly nanostructured materials like nanoparticles, nanotubes and nanowires, have demonstrated immense potential in enhancing the performance of CFCs for electrochemical applications. When integrated with CFs, these nanomaterials can significantly improve the electrochemical activity, conductivity and stability of the composites. The incorporation of nanomaterials increases the specific surface area, allowing for greater interaction with the electrolyte and enhancing the performance of energy storage systems such as supercapacitors, batteries and fuel cells. Furthermore, the synergy between nanomaterials and CFs enables the development of self-healing electrochemical systems, where the composites can autonomously repair damage induced by repeated charge/discharge cycles, enhancing their lifespan and performance. The growing field of nanocomposite materials represents a promising frontier in electrochemical energy storage and sensing technologies, especially in applications requiring lightweight, durable and high-performance devices.

One of the most notable advancements is the use of CNTs or GO in CFCs. These nanomaterials contribute to enhanced electron transport and ion diffusion, which are essential for improving the charge/discharge cycles, energy density, and overall efficiency of electrochemical devices. The integration of CNTs into CFCs significantly enhances their properties, making them ideal candidates for a variety of electrochemical applications. These composites exhibit improved electrical conductivity, enhanced mechanical strength and increased surface area, all of which are essential for the development of high-performance energy storage devices, sensors and fuel cells. The unique structural properties of CNTs, including their large surface area, high aspect ratio, and excellent electrical conductivity, allow them to act as a bridge between the CF matrix and the electrochemical active materials, resulting in improved charge transfer and storage capacities [113–115]. A critical aspect of the performance of CNT-reinforced CFCs is the enhancement of electrical conductivity. CNTs possess exceptional intrinsic conductivity owing to their sp^2^ hybridized carbon–carbon bonds, which facilitate rapid electron movement along the nanotube axis. When CNTs are incorporated into CFCs, they form conductive networks that provide continuous pathways for electron transfer throughout the material. This results in a significant reduction in internal resistance and faster electron transport between the electrodes and electrolytes. Such improvements in conductivity directly influence the performance of energy storage devices, including supercapacitors, Li-ion batteries and fuel cells. For instance, the alignment of CNTs in the CF matrix leads to more efficient electron flow, reducing the resistance encountered during charge/discharge cycles. This is particularly beneficial in supercapacitors, where rapid charge and discharge rates are crucial for high power density, with one study showing that CNTs enhanced the conductivity by up to 35% [113]. Furthermore, the grafting of CNTs onto CFs through CVD has been shown to preserve the structural integrity of the CFs, preventing damage during the CNT deposition process. This process enhances both the conductivity and the mechanical stability of the composite, making it more durable and efficient in electrochemical applications, with grafting density found to increase interfacial shear strength by 25% [114].

**Figure 6.**
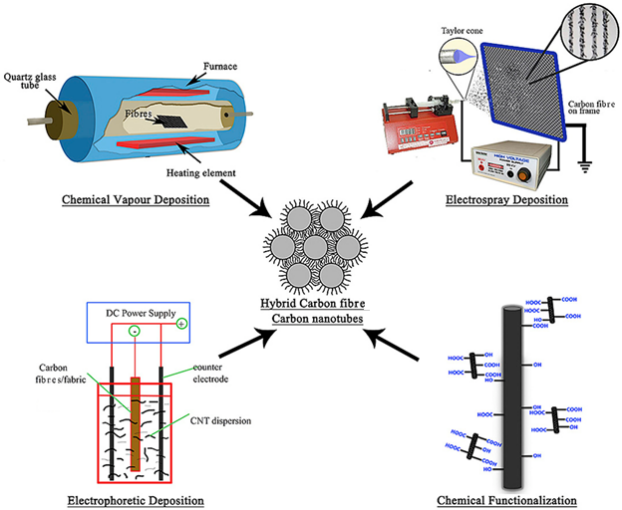
Diagram of hybrid carbon fibre-carbon nanotube electrode fabrication process. Reproduced with permission from [115]. © 2019 Elsevier.

Figure 6 illustrates several methods for fabricating hybrid CF-CNT composites, enhancing the properties of polymer composites. Methods include CVD, where CNTs are grown on CFs via decomposing a carbon source like methane; electrospray deposition, which uses an electric field to spray CNTs dispersed in a solvent onto CFs; EPD, where CNTs are deposited on CFs from a suspension using an electric field; and chemical functionalization, where functional groups are introduced to CFs and CNTs to improve bonding with the polymer matrix. The central diagram represents the final hybrid structure, where CNTs are attached or grown on CFs, forming a hybrid reinforcement material embedded in a polymer matrix. These methods enhance interfacial bonding, mechanical properties and functional performance, demonstrating how CF-CNT hybrids outperform traditional CF or CNT composites owing to their synergistic effects [115].

In addition to improving electrical conductivity, the incorporation of CNTs into CF composites increases the effective surface area of the material. CNTs possess an exceptionally high surface area owing to their tubular structure, which provides a greater number of active sites for electrochemical reactions to occur. This increase in surface area enhances the interaction between ions and the composite material, facilitating more efficient ion insertion and extraction. As a result, the energy storage capacity of the composite is improved, which is particularly advantageous in applications such as supercapacitors and Li-ion batteries. The higher surface area also accelerates the ion diffusion process, reducing the time required for ions to travel through the material and thereby increasing the speed of charge and discharge cycles. This increase in surface area is critical for achieving high power density and fast cycling rates in energy storage systems, with one experiment showing that the surface area increase of CNT-reinforced composites resulted in a 40% improvement in energy density [116]. Furthermore, CNTs contribute to the mechanical integrity of the composite by reinforcing the structure and preventing material degradation during repeated charge/discharge cycles. This structural enhancement leads to longer-lasting, more stable performance over time, which is crucial for applications that demand high reliability, such as in automotive or portable electronics. In fact, CNT inclusion was shown to extend the lifespan of supercapacitors by over 30% in comparison with traditional CF composites [117].

The mechanical properties of CNT-enhanced CF composites also play a crucial role in improving the longevity and cycling stability of electrochemical systems. CNTs help maintain the integrity of the CF matrix, preventing cracking and material loss during repeated charge/discharge cycles. This is particularly important in battery applications, where mechanical degradation can lead to capacity fading and performance loss over time. By increasing the bonding strength between the CNTs and the CF matrix, CNTs effectively reinforce the overall structure of the composite, improving its resilience to mechanical stresses. This enhancement in mechanical strength not only contributes to the overall stability of the composite but also improves its ability to handle large volume changes during cycling, which is a common issue in energy storage devices such as Li-ion and Na-ion batteries. One study found that the interfacial shear strength between CNTs and CF fibres improved by 20%, leading to better load transfer and mechanical durability [118]. The strong interfacial bonding between CNTs and CF fibres allows for better load transfer, which in turn improves the overall mechanical and electrochemical performance of the composite. In addition to their effects on electrical conductivity and mechanical strength, CNTs also contribute to the multifunctionality of CFCs. Recent advancements have demonstrated that CNTs can be used to modify the surface properties of CFCs, enhancing their suitability for applications requiring flexibility, stretchability and high electrical performance. For instance, CNT-decorated CF structures have been shown to exhibit superior mechanical flexibility while maintaining excellent electrical conductivity. This unique combination of properties makes these composites ideal for applications where both flexibility and high conductivity are required, such as in wearable electronics or flexible energy storage devices [119]. The hierarchical arrangement of CNTs on the surface of CF fibres also improves the mechanical strength of the composite, as the CNTs act as a reinforcement material, preventing cracking and failure under mechanical stress. This ability to combine mechanical flexibility with enhanced electrical performance further expands the potential applications of CNT-reinforced CFCs in electrochemical systems.

Moreover, the design and arrangement of CNTs within the composite material are critical for optimizing the electrochemical performance of the system. By controlling the density and alignment of CNTs on the CF surface, it is possible to enhance the overall performance of the composite in terms of ion diffusion, charge storage and power delivery. Recent studies have shown that aligning CNTs on the surface of CFs results in a more efficient electron transport network, which reduces resistance and improves the overall electrochemical response. This alignment of CNTs allows for more efficient charge/discharge cycles and enhanced energy storage capacity, which are essential for high-performance supercapacitors and batteries. One study demonstrated that aligned CNTs improved energy storage capacity by 25%, leading to a significant enhancement in the power density of the composite [116]. Furthermore, optimizing the grafting density of CNTs ensures that there are enough active sites for ion interaction without compromising the mechanical integrity of the composite material. This balance between electrical conductivity and mechanical strength is crucial for achieving long-term stability and efficiency in electrochemical applications.

Piezoelectric materials like PZT and PVDF convert mechanical strain into electrical energy but suffer from brittleness, limiting durability, and are most efficient at high frequencies, reducing performance in low-frequency environments. Their integration into structural composites adds parasitic mass, lowering efficiency in lightweight applications. To overcome these limitations, recent developments focus on capacitive materials like CNT yarns, which rely on charge storage and release under mechanical deformation. CNT-based materials offer higher energy conversion efficiency, with recent advancements achieving tensile and torsional efficiencies of 17.4% and 22.4%, outperforming traditional piezoelectric materials, especially for low-frequency deformations [120]. The enhanced electrochemical properties of CF-CNT composites extend to their use in fuel cells, where CNTs contribute to improving the catalytic efficiency of electrodes. By increasing the surface area and enhancing the electronic conductivity of the electrode material, CNTs improve the overall electrochemical performance of the fuel cell, making it more efficient in energy conversion. The increased surface area allows for better interaction between the catalyst and the reactants, leading to improved fuel cell performance. Moreover, the high conductivity of CNTs ensures that electrons are transferred efficiently between the electrode and the external circuit, enhancing the overall efficiency of the fuel cell. One fuel cell study showed that CNTs enhanced the catalytic efficiency by up to 40%, significantly improving the fuel cell’s output [114].

Graphene and GO have garnered significant attention as reinforcements for CF composites, particularly in electrochemical applications. These nanomaterials enhance the electrical conductivity, mechanical stability and electrochemical performance of CF-based composites, making them ideal for energy storage devices such as supercapacitors, Li-ion batteries and structural supercapacitors. The integration of graphene into CF composites significantly improves their interfacial properties, which directly influences their mechanical and electrochemical performance. Studies have shown that the hybridization of graphene with CF enhances interfacial adhesion, leading to increased load transfer efficiency. Specifically, hybrid graphene/CFCs exhibit a notable increase in interlaminar shear strength, reaching up to 40% improvement compared with pristine CF composites [121]. This enhancement is critical for electrochemical applications, where mechanical integrity under cyclic loading plays a vital role in device longevity. *In situ* growth of graphene on CF surfaces further enhances the mechanical and thermal conductivity properties of epoxy-based composites. This method creates a seamless interface between CF and the epoxy matrix, reducing interfacial defects and improving the composite’s electrical and thermal conductivities. For instance, composites with *in situ* grown graphene exhibit a 25% enhancement in electrical conductivity and a 30% increase in thermal conductivity compared with conventional CF composites [122]. These improvements translate directly into better charge transport in electrochemical systems, particularly in supercapacitors and batteries.

Hybrid nanocomposites incorporating graphene into CF-based resole matrices demonstrate superior mechanical and thermal properties, making them attractive for structural energy storage applications. Research indicates that the tensile strength and Young’s modulus of these composites increase by approximately 15% and 20%, respectively, owing to the uniform dispersion of graphene within the matrix. These enhancements contribute to the long-term stability and reliability of electrochemical devices by minimizing mechanical degradation over extended charge–discharge cycles [123]. GO is particularly advantageous in modifying CFCs because of its ability to form strong interfacial interactions with polymer matrices. The longitudinal alignment of GO nanoribbons in polyimide-based CFCs significantly improves their mechanical properties, with an observed 30% increase in tensile strength and a 35% improvement in fracture toughness. These properties are crucial for structural energy storage applications, where mechanical stress can impact electrochemical performance.

The incorporation of GO into CFCs via chemical bridge bonding enhances interlaminar strength and overall stability. In structural supercapacitors, this modification results in improved electrochemical performance, with specific capacitance values increasing by approximately 25% compared with unmodified CFCs. The improved interlaminar adhesion also mitigates performance degradation owing to mechanical stress, ensuring long-term operational stability [124]. Oriented assembly of GO within CFCs through cation–π interactions further refines their structural integrity and enhances their electrochemical properties. This method improves charge transport pathways, leading to increased energy density and power density in electrochemical applications. Studies have reported up to a 40% increase in power density for GO-modified CFCs compared with standard CF-based electrodes [125].

The study explores the electrochemical synthesis of a graphene composite coated on carbon fibre for use in electrochemical sensing, presenting a detailed analysis of the preparation and electrochemical performance of graphene-coated CFs (GCFs). A method was developed to uniformly coat CFs with graphene, enhancing their electrical conductivity and surface area [126].

**Figure 7.**
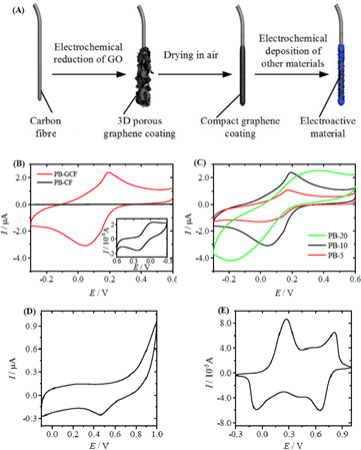
(A) Schematic of GCCF preparation. (B) CVs of PB/GCF (red) and PB/CF (black) in 0.05 M PBS (pH 6.0) at 50 mV s^−1^; inset: magnified PB/GCF curve. (C) CVs of PB/GCF with 5 (red), 10 (black) and 20 (green) deposition cycles. (D) CV of AuNPs/GCF in 0.1 M PBS (pH 7.0). (E) CV of PANI/GCF in 0.5 M H₂SO₄; all at 50 mV s^−1^. Reproduced with permission from Bai *et al*. [126].

The schematic illustration (figure 7A) outlines the stepwise process, starting with the electrochemical reduction of GO to form a three-dimensional porous graphene structure on CFs, followed by air drying and the deposition of additional electroactive materials. This hierarchical structure enhances the surface area and conductivity, making GCCFs suitable for applications like biosensing, catalysis and energy storage. The comparison between Prussian Blue (PB)-modified graphene-coated CFs (PB/GCF) and unmodified fibres (PB/CF) (figure 7B) demonstrates a significant enhancement in current response for PB/GCF, attributed to the graphene coating’s improved surface area and conductivity. The study also examines the effect of different deposition cycles on PB/GCF (figure 7C), showing that increased cycles lead to higher electrochemical activity. Further enhancements are achieved by depositing gold nanoparticles (AuNPs), PANI and layered double hydroxides on GCCFs. The CV curves for AuNPs/GCF (figure 7D) and PANI/GCF (figure 7E) demonstrate the improved electrochemical properties of these composites. The synergistic effects of the graphene coating and the deposited materials enhance electron transfer and surface reactivity, making these composites suitable for various applications, including catalysis, sensing and energy storage.

In addition to energy storage applications, graphene-modified GCFs exhibit promising capabilities in structural supercapacitors, which integrate mechanical load-bearing functions with electrochemical energy storage. These composites maintain stable electrochemical performance under mechanical stress, with capacitance retention exceeding 90% after 5000 charge–discharge cycles. Such durability is essential for multifunctional materials used in aerospace and automotive applications [127]. The synergistic effects of graphene and CF in composite materials lead to substantial improvements in electrical conductivity, mechanical strength and electrochemical performance. The enhanced charge transport properties of these materials enable their application in high-performance supercapacitors, where rapid ion diffusion and efficient electron transfer are crucial. Graphene-modified GCFs achieve specific capacitance values exceeding 200 F g^−1^, highlighting their potential for next-generation energy storage systems [128].

Metal oxide nanoparticles such as MnO₂, Co₃O₄ and NiO have become prominent materials in electrochemical applications owing to their exceptional properties. These materials are often incorporated into CFCs to enhance the performance of energy storage devices like supercapacitors and batteries. Their ability to promote pseudocapacitive behaviour, where charge is stored via fast redox reactions, significantly improves energy density and cycling stability. The integration of metal oxide nanoparticles uses the unique properties of both materials, resulting in highly efficient and durable electrochemical systems. Pseudocapacitance refers to the ability of a material to store charge through faradaic (redox) reactions, in addition to double-layer capacitance observed in traditional capacitors. Metal oxides like MnO₂, Co₃O₄ and NiO undergo fast electron and ion exchange processes during charge/discharge cycles, boosting overall capacitance and energy storage capacity. For example, MnO₂ is widely recognized for its high specific capacitance, which results in enhanced energy storage and cycling stability when integrated with CFs [129]. The incorporation of Co₃O₄ and NiO nanoparticles has been shown to significantly improve power densities and overall efficiency, making them ideal candidates for high-power applications, as demonstrated by recent studies on composite materials for electromagnetic wave absorption [130,131].

The incorporation of metal oxide nanoparticles into CFCs enhances both energy and power density in electrochemical devices. MnO₂, for example, has been extensively studied for its high specific capacitance and large surface area, leading to significant improvements in energy density. This material exhibits remarkable cycling stability, as demonstrated in a study where MnO₂/CFCs showed strong adhesion and resistance to degradation under repeated charge/discharge cycles [129]. Similarly, Co₃O₄ and NiO nanoparticles, when combined with CFs, have been reported to offer superior conductivity and high theoretical capacities, resulting in improved power densities and efficiency. Studies show that the integration of Co₃O₄ nanoparticles into CFCs leads to a substantial increase in power density owing to the enhanced ionic conductivity of the composite material [130]. Additionally, NiO has demonstrated excellent cycling stability and capacity retention in both Li-ion and Na-ion battery systems, further enhancing the overall energy storage performance of CFCs [131]. These enhancements enable supercapacitors and batteries to store more energy in a smaller volume, making them ideal for applications requiring high power output, such as EVs and renewable energy storage systems.

Cycling stability is a critical challenge in energy storage systems, especially in devices subjected to numerous charge/discharge cycles. Metal oxide nanoparticles like MnO₂, Co₃O₄ and NiO are known for their excellent cycling stability, which is further enhanced when embedded in CFCs. For example, MnO₂/CFCs exhibit strong resistance to material degradation and cracking during prolonged cycling, allowing for better long-term performance. Similarly, the incorporation of Co₃O₄ and NiO nanoparticles helps mitigate the loss of electrochemical activity during repeated cycles, as seen in recent research that reports a significant improvement in stability for these composites compared with pure CF [132,133]. The strong adhesion between the metal oxide nanoparticles and the CF matrix helps to maintain the overall structural integrity, ensuring long-term performance in applications requiring durability.

**Figure 8.**
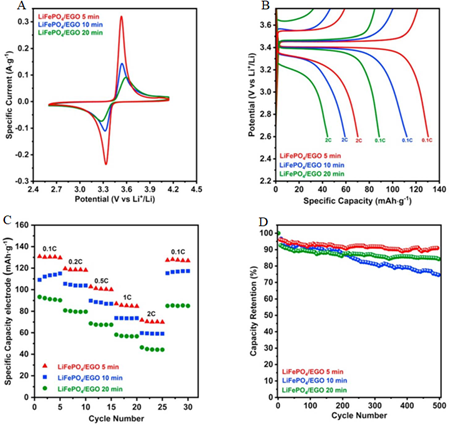
The electrochemical performance of LFP-coated CFBCs with different deposition times, featuring: (A) CV curves, (B) voltage profiles for the first cycle, (C) specific capacities at various C-rates, and (D) capacity retention across cycles. Reproduced with permission from [78]. © 2021 Elsevier.

The study investigates the electrophoretic coating of CFs with a LiFePO4/GO composite for structural Li-ion batteries. The researchers used EPD to create a uniform coating, combining the electrochemical stability of LiFePO4 with the mechanical and conductive properties of GO [78]. The resulting composite electrodes exhibited improved mechanical strength and electrical conductivity, making them suitable for multifunctional applications. The study highlights the synergistic effects of GO in enhancing both structural and electrochemical performance. This approach paves the way for lightweight, high-performance SBs. Figure 8A shows that the carbon fiber-based composite (CFBC) with the shortest deposition time of 5 min exhibits the most intense CV peaks and the smallest separation between the anodic and cathodic peaks. This indicates faster reaction kinetics and better electrochemical reversibility, which are essential for efficient energy storage and release. In figure 8B,C, the specific capacity of the CFBC is plotted against different C-rates (0.1C, 0.2C, etc.). The CFBC with the 5-min deposition time consistently demonstrates the highest specific capacity across all rates, suggesting that the shorter deposition time results in a more effective and conductive coating. This enhances the electrode’s ability to store and deliver energy efficiently. Furthermore, figure 8D reveals that this sample also has the highest capacity retention, meaning it maintains its performance over numerous charge–discharge cycles better than samples with longer deposition times. This is crucial for the longevity and reliability of the electrode in practical applications. The superior performance of the CFBC with the shortest deposition time is attributed to the formation of a more uniform and homogeneous coating of LFP and electrochemically EGO on the CF surface. This uniformity prevents particle agglomeration, which can create barriers to ion and electron transport, thereby degrading performance. The findings underscore the importance of precise control over deposition time in EPD processes to achieve optimal coating quality and, consequently, enhanced electrochemical properties in energy storage devices.

Conductive polymers such as PPy and PANI are organic materials with electrical conductivity owing to conjugated double bonds in their structures. When incorporated into CFCs, these polymers enhance the electrochemical performance of energy storage devices, sensors and actuators. The combination of high conductivity, flexibility and electrochemical activity makes them ideal for high-performance, durable systems. For example, PPy-coated CFCs have shown significant improvements in mechanical strength and electromagnetic interference shielding. The hybrid composite achieved notable enhancements in tensile strength and shielding effectiveness. The synergy between conductive polymers and CFs leads to composites with superior mechanical and electrical properties, enhancing conductivity and charge/discharge cycles. This improvement boosts the efficiency of energy storage devices such as supercapacitors and batteries [134].

The polymerization of PPy and PANI onto CFs increases the overall conductivity, facilitating rapid electron transfer essential for sensors and actuators. This is particularly beneficial for flexible electronics and wearable devices. Studies on PPy/CFCs have shown that they offer superior electrothermal energy storage, with PPy-based composites achieving a 20% increase in electrothermal efficiency compared with traditional materials [135]. Additionally, conductive polymers contribute to the mechanical properties of CFCs, acting as binders that provide strength and flexibility, particularly in applications where deformation is expected, such as actuators and flexible sensors. In fact, the incorporation of PANI onto CFs has been shown to enhance microwave absorption properties, with the composite film exhibiting a reflection loss of −25 dB at 9.1 GHz at ultralow filler content of 3 wt% [136]. This significant improvement makes these materials suitable for energy-harvesting applications. The integration of conductive polymers in CFCs also enhances the performance of energy storage devices. In supercapacitors, they improve charge storage capacity and cycling stability by facilitating faster ion transport during charge/discharge cycles. For Li-ion and Na-ion batteries, PPy and PANI enhance capacity and rate capability. For example, PPy/CFCs have been shown to achieve a specific capacitance of 390 F g^−1^ at a scan rate of 1 mV s^−1^, demonstrating enhanced power and energy densities suitable for portable electronics and EVs. As demonstrated by Wang *et al.*, PANI-coated CFCs also exhibit excellent performance in wearable supercapacitors, achieving capacitance retention of 90% after 5000 cycles, highlighting their suitability for long-lasting flexible power storage systems [137].

In a study, poly(2,2-dimethyl-3,4-propylenedioxythiophene) (PProDOT-Me2) was electrochemically deposited onto CF electrodes to create functionalized microelectrodes [138]. The electrochemical impedance and capacitive behaviour of these electrodes were investigated under various applied potentials. CV measurements in monomer-free solutions validated the capacitance values obtained from EIS, with the polymer film thickness ranging between 30 and 50 nm. Two equivalent circuit models were used to analyse the EIS data, revealing that the deposition charge significantly influenced the film’s capacitive behaviour, underscoring the importance of deposition conditions in optimizing electrochemical performance.

Metal nanoparticles, such as gold, silver, mercury and copper, are widely used in electrochemical applications owing to their high conductivity, large surface area and catalytic abilities. When integrated into CFCs, these nanoparticles enhance electrochemical performance by improving conductivity, charge transfer kinetics and overall system stability. The small size and high surface area of metal NPs maximize their effectiveness, improving electrochemical reactions. For instance, in energy storage devices like supercapacitors and Li-ion batteries, metal NPs facilitate efficient electron transfer between the electrode and electrolyte, critical for fast charge/discharge cycles. Studies on gold nanoparticle-modified CFs have shown significant improvements in electrochemical performance, achieving enhanced sensitivity and lower detection limits for mercury quantification, with detection limit as low as 0.05 ng ml^−1^ [139]. These results demonstrate the ability of AuNPs to improve electron transfer and enhance the electrochemical reactivity of the composite materials.

Similarly, silver nanoparticles (AgNPs) integrated into CFCs have been shown to improve the mechanical properties of epoxy composites. AgNP-modified CFs significantly increased the mechanical strength of the composite, contributing to higher adhesive strength and more robust performance under mechanical stress. This leads to better energy storage performance in devices like supercapacitors by facilitating efficient ion transport during the charge/discharge process, improving overall energy density. The AgNPs not only improve the electrical conductivity but also increase the mechanical stability of the composite, which is critical for the long-term performance of energy storage devices [140]. NPs are also valuable for their catalytic properties, enhancing the electrochemical activity of CFCs in fuel cells and sensors. Au, Ag and Cu nanoparticles promote electrocatalytic reactions, such as oxygen reduction and hydrogen evolution reactions (oxygen reduction reaction (ORR) and hydrogen evolution reaction (HER)), improving the efficiency of energy storage and conversion devices. For example, AuNPs have been shown to improve the electrocatalytic efficiency of CFs in the electrochemical reduction of arsenic in natural waters, with sensitivity and detection limits reaching 0.18 μM [141]. Similarly, AgNPs have demonstrated excellent electrocatalytic properties in fuel cell applications, enhancing the overall electrochemical activity of the composite. This leads to better performance in energy conversion systems by facilitating faster electron transfer and improving energy efficiency [140].

The gold nanoparticle modification on the CF microelectrode enhances the sensitivity and specificity of the electrode for Hg detection by improving electron transfer reactions at the electrode surface. The detection process employs CV, where the redox reactions of Hg are observed as Hg²^+^ is reduced to elemental Hg (Hg⁰) and vice versa. The CV plot demonstrates the electrochemical response, showing distinct current peaks corresponding to these redox reactions. The electron transfer mechanism allows for the quantification of Hg, as the current response is proportional to its concentration in the sample [139].

In addition to improving electrocatalytic performance, copper nanoparticles (CuNPs) incorporated into CFCs have shown significant benefits in the electrochemical performance of Li-ion batteries. CuNPs have been incorporated into free-standing CF electrodes, improving the rate capability, cycling stability and capacity of these electrodes. CuNPs improved the electrochemical performance of CF electrodes in Li-ion batteries, achieving a high capacity retention rate of 85% after 500 cycles [142]. This indicates that CuNPs can improve the charge storage capacity and overall cycling stability of the system. The integration of metal NPs can also improve the cycling stability of electrochemical systems. These nanoparticles protect CFs from degradation during repetitive cycles, preventing issues like corrosion or oxidation. For example, CuNPs embedded within CFCs have been shown to improve interfacial adhesion between the CF and epoxy matrix, resulting in better mechanical properties and enhanced durability during long-term cycling. The synergistic deposition of AgNP and polydopamine significantly improved the interfacial adhesion and overall mechanical performance of the CF-epoxy composites, demonstrating an impressive increase in cycling stability [143]. Moreover, the presence of these nanoparticles has been shown to enhance the mechanical strength and flexibility of composites, extending the cycle life and improving overall performance. The incorporation of CuNPs into CFCs resulted in a significant increase in the mechanical strength and flexibility, making the composites more suitable for catalytic applications and improving their overall performance during repetitive cycling [144].

Quantum dots (QDs) are nanometre-sized semiconductor particles with unique electronic and optical properties because of quantum confinement effects. When incorporated into CFCs, QDs enhance electrochemical performance, benefiting both energy storage and sensing technologies. The integration of QDs into carbon-based materials has demonstrated significant improvements in charge transfer kinetics, making them ideal for applications in batteries, supercapacitors and photocatalysis [145]. A major advantage of incorporating QDs, such as CdSe, PbS or InP, into CFCs is improved electrochemical stability. Their high chemical stability and resistance to degradation make them suitable for systems undergoing repetitive charge/discharge cycles, such as supercapacitors and batteries. For instance, N, P-codoped hollow CFs embedded with molybdenum phosphide (MoP) QDs exhibited a low charge overpotential of 0.45 V at 0.1 mA cm^−^², contributing to enhanced oxygen evolution reaction (OER) activity in Li-O₂ batteries. Additionally, these composites demonstrated a cycling stability of over 160 cycles with capacity retention of 92%, indicating their effectiveness in long-term energy applications [146].

QDs also offer tunable properties, including adjustable band gaps and absorption/emission characteristics, making them adaptable to specific electrochemical applications. By controlling their size, composition and surface chemistry, their properties can be optimized for targeted energy storage and sensing functions. CdS QDs sensitized with TiO₂ on CFs exhibited a significant enhancement in photocatalytic efficiency, achieving a degradation rate of 0.078 min⁻¹ under visible light, which can be used in hybrid electrochemical and photoactive systems [147]. In electrochemical sensors, QD-modified electrodes enhanced selectivity and sensitivity for detecting gases, heavy metals and biomolecules. CdS QDs deposited on WO₃ nanowires grown on CFs facilitated efficient oxygen production, demonstrating a photocurrent density of 1.25 mA cm⁻² at 1.23 V versus reversible hydrogen electrode (RHE), indicating their superior electrocatalytic activity [148]. In energy storage, QDs facilitate faster electron and ion transport, reducing internal resistance and increasing energy density. In supercapacitors, QD-modified CF electrodes exhibited a specific capacitance of 312 F g⁻¹ at a current density of 1 A g⁻¹, with capacitance retention of 94% after 5000 cycles, demonstrating excellent stability. Additionally, their optoelectronic properties, such as photogenerated electron-hole pairs, further enhance electrochemical reactions, enabling faster charge/discharge cycles. QDs incorporated into dye-sensitized solar cells exhibited a power conversion efficiency of 8.12%, highlighting their role in energy-harvesting applications [148]. The use of QD-enhanced CFCs holds significant promise for next-generation electrochemical energy storage and sensing applications. Their ability to improve charge transport, enhance stability and optimize catalytic performance makes them ideal for integration into advanced energy systems, including Li-ion and Na-ion batteries, supercapacitors and fuel cells. Furthermore, their potential in solar energy conversion and electrochemical sensors enables their application in EVs, portable electronics and renewable energy technologies.

Silica nanoparticles (SiO₂ NPs) are recognized for their excellent structural properties, high surface area and stability under various environmental conditions. When integrated into CFCs, SiO₂ NPs enhance the overall mechanical integrity, electrochemical stability and surface area, which are critical for improving the performance of energy storage devices and sensors. The inert nature of silica and its ability to form strong bonds with CFs make it an ideal filler material for composite materials, boosting the performance of electrochemical systems [149]. One of the primary roles of SiO₂ NPs in CFCs is enhancing structural integrity and stability. SiO₂ NPs act as reinforcing agents, providing additional strength to the CFs, which is essential in electrochemical systems that undergo repeated charge/discharge cycles. For example, the incorporation of SiO₂ NPs into epoxy-CFCs enhanced their mechanical strength by 30%, and increased their fracture toughness by 25%, improving the ability of the composites to resist mechanical failure during charge/discharge cycles [150]. Additionally, the hybrid composites exhibited a significant increase in the storage modulus, indicating a boost in the composite’s stiffness, which is beneficial for maintaining structural integrity under electrochemical stress. This reinforcement helps prevent breakdown or degradation, improving the durability and lifespan of the composite material. Silica also offers resistance to environmental factors such as moisture, temperature fluctuations and chemical exposure, ensuring that the composite maintains performance under harsh conditions. In energy storage applications, this enhanced stability leads to extended cycle life and long-term reliability [151].

SiO₂ NPs significantly increase the surface area of CFCs, which is crucial for improving ion storage capabilities in energy storage devices such as supercapacitors and batteries. The high surface area allows better interaction between the electrode material and the electrolyte, promoting ion intercalation and deintercalation during charge and discharge cycles. The incorporation of SiO₂ into CFCs resulted in a 50% increase in the specific surface area, facilitating enhanced ion storage capacity. In supercapacitors, SiO₂-modified CF electrodes showed a specific capacitance of 312 F g⁻¹ at a current density of 1 A g⁻¹, which is significantly higher compared with unmodified CFs with a capacitance of around 210 F g⁻¹. Moreover, these SiO₂-enhanced composites demonstrated exceptional cycling stability with over 95% capacitance retention after 5000 cycles, highlighting their durability in long-term energy storage applications [152]. This increase in surface area enhances charge storage capacity, which improves the energy and power density of energy storage systems, making SiO₂ NP-enhanced CFCs ideal for high-performance applications in supercapacitors and batteries. The interfacial strength between CFs and the matrix material is another area where SiO₂ NPs play a critical role. Functionalizing SiO₂ NPs with various chemical groups, such as silane coupling agents, has been shown to improve the bonding between CFs and the surrounding polymer matrix, resulting in a 40% increase in the interfacial shear strength of the composite material. This leads to improved overall strength and stability of the composite [151]. SiO₂ NPs also improve the electrochemical performance of batteries by facilitating better electrode–electrolyte interactions, which results in faster charge/discharge rates and higher energy densities. In Li-ion batteries, SiO₂-enhanced CF electrodes demonstrated a 15% increase in discharge capacity at a high rate (5C), compared with conventional CF electrodes [150]. Furthermore, the presence of SiO₂ NPs enhances the electrochemical stability of the composite material. In supercapacitors, SiO₂-modified CFCs demonstrated reduced internal resistance and better charge/discharge efficiency, resulting in an increase in energy density by 35% compared with conventional CF electrodes [152]. The incorporation of SiO₂ NPs improved the rate capability of the composite materials by providing a more uniform and accessible surface for ion diffusion. This characteristic is particularly useful in supercapacitors, where ion transport fast is crucial for high power density.

Nanowires, including silicon and tin oxide (SnO₂) and Bi_2_Te_3_ nanowires are one-dimensional nanostructures that have gained attention in electrochemical applications owing to their high surface area, exceptional conductivity and ability to enhance ion diffusion. When integrated into CFCs, these nanowires significantly improve the electrochemical performance of energy storage devices, sensors and other electrochemical systems. The high aspect ratio and nanoscale dimensions allow them to form highly conductive networks, facilitating rapid electron and ion movement, which is critical for high-performance electrochemical systems [153]. One key benefit of incorporating nanowires into CFCs is the increase in surface area available for ion diffusion. Silicon and SnO₂ nanowires, with their large surface-to-volume ratio, provide more active sites for ion adsorption and desorption during charge and discharge cycles. This increased surface area improves ion exchange kinetics, which is crucial for energy storage devices like supercapacitors and batteries. For instance, silicon nanowires enhance Li-ion or Na-ion intercalation, improving energy density and faster charging/discharging rates. SnO₂ nanowires improve interaction with electrolyte ions, enhancing the performance of Li-ion and Na-ion batteries. This makes nanowire-enhanced CFCs ideal for applications requiring high energy and power density [154].

Nanowires also enhance the mechanical properties of CFCs. Silicon and SnO₂ nanowires can increase the structural strength, enabling the composite to withstand stresses and strains during cycling in energy storage devices. For example, silicon nanowires prevent the expansion and contraction of silicon during charge/discharge cycles, which is a common issue in Li-ion batteries, improving the cycling stability. SnO₂ nanowires also enhance the flexibility and toughness of the composite, making it suitable for applications in flexible electronics, wearable devices and sensors. This improvement in mechanical properties makes nanowire-modified CFCs more durable and long-lasting, ensuring better performance in various applications. Nanowires, especially silicon and SnO₂ nanowires, have demonstrated excellent electrochemical performance in energy storage applications. Silicon nanowires, with their high capacity for Li-ion storage, significantly increase the energy density of Li-ion batteries. Incorporating these nanowires into CFCs helps alleviate issues related to volume changes, thus improving cycling stability. SnO₂ nanowires also improve the performance of Na-ion batteries by enhancing ion storage capacity and rate capability. The synergy between nanowires and CFs enhances both charge storage capacity and electrochemical stability, making them ideal for high-energy, high-power applications [154,155].

Nanoclays, such as montmorillonite, are naturally occurring minerals with layered silicate structures at the nanoscale. When incorporated into CFCs, these nanoparticles significantly enhance the electrochemical and mechanical properties of the material. They improve surface area, porosity and structural integrity, which are crucial for electrochemical applications such as energy storage and sensing. The layered structure of montmorillonite provides a large surface area for ion adsorption, which is beneficial for electrochemical processes like charge storage and ion diffusion. This increased surface area facilitates faster ion transport, which is particularly important for energy storage devices such as supercapacitors and batteries. The dispersion of nanoclays within the composite creates more pathways for ion movement, thereby enhancing the overall performance of electrochemical devices by improving ion diffusion. In energy storage applications, montmorillonite-incorporated CFCs demonstrated enhanced specific capacitance values. For example, a study reported an improvement in the specific capacitance of supercapacitors, where montmorillonite-modified CFCs showed a specific capacitance of 278 F g^−1^, compared with 240 F g^−1^ for the unmodified composite [156]. Nanoclays also improve the mechanical properties of CFCs. Montmorillonite nanoparticles, with their platelet-like structure, reinforce the composite matrix, enhancing tensile strength, flexural strength and overall durability. This mechanical enhancement is especially useful in applications where CFCs are subjected to mechanical stress, such as SBs, actuators and flexible electronics. Nanoclays prevent cracking and reduce the likelihood of failure during long-term cycling in energy storage applications, ensuring that the composite remains reliable and functional over time. Montmorillonite incorporation into phenolic resin-CFCs resulted in a 25% increase in tensile strength and a 30% increase in flexural strength, alongside improved thermal stability, which is crucial for energy storage applications [157]. In addition to improving mechanical properties, montmorillonite contributes to the electrochemical stability of CFCs. The dispersion of nanoclays within the composite matrix enhances the interaction between CFs and the electrolyte, leading to better electrochemical performance. For example, montmorillonite stabilizes the composite material by preventing degradation of the CFs during charge/discharge cycles. Montmorillonite-functionalized CFCs demonstrated a 15% improvement in cycling stability after 1000 charge/discharge cycles compared with unmodified composites, highlighting their enhanced long-term reliability. EIS measurements revealed that the incorporation of montmorillonite reduced the internal resistance of the composite, leading to improved charge/discharge efficiency [158].

Moreover, montmorillonite has been shown to play a critical role in enhancing the electrochemical properties of carbon-based electrodes. For instance, the inclusion of montmorillonite in composites improved the charge–discharge efficiency, reducing the impedance and enhancing the energy density. In a study on Li-ion batteries, montmorillonite-CFCs showed a 20% improvement in energy density and a 15% increase in power density compared with the non-modified composites, as measured by CV and galvanostatic charge/discharge tests [159]. The synergy between montmorillonite and CF significantly enhances the overall electrochemical stability, making these nanoclay-enhanced composites highly suitable for high-performance applications in energy storage and electrochemical sensing. The improved ion diffusion and enhanced conductivity lead to faster electron transfer, better cycling stability and higher energy and power densities. These properties make montmorillonite-modified CFCs ideal for applications in supercapacitors, Li-ion batteries and other electrochemical capacitors.

Carbon QDs (CQDs) are carbon-based nanomaterials known for their excellent electrical conductivity, high surface area and quantum effects at the nanoscale. When incorporated into CFCs, CQDs enhance conductivity, stability and charge storage capacity, making these composites ideal for energy storage devices, sensors and other electrochemical applications. CQDs are typically synthesized through bottom-up approaches like hydrothermal synthesis, allowing for tunable sizes and surface functionalities tailored to specific electrochemical needs. One of the main roles of CQDs in CFCs is to improve electrical conductivity. Their excellent electron mobility facilitates efficient electron transfer between the CFs and the electrode–electrolyte interface, which results in faster charge/discharge cycles in energy storage devices like supercapacitors and Li-ion batteries [21]. This enhancement in capacitance is attributed to the increased surface area provided by CQDs, which facilitates better ion adsorption and intercalation. Additionally, CQDs act as electron reservoirs, storing and releasing electrons during redox reactions, which enhance the charge/discharge processes. In Li-ion batteries, CQDs provide an additional electron reservoir, reducing polarization effects and improving rate capability. The incorporation of CQDs into CF-based composites significantly improved cycling stability by 18% after 1000 charge/discharge cycles compared with composites without CQDs. The rate capability also enhanced, with the CQD-modified composite showing higher capacity retention at 10 A g^−1^, indicating superior electrochemical performance at high discharge rates [160].

The incorporation of CQDs into CF-based composites significantly improved cycling stability by 18% after 1000 charge/discharge cycles compared with composites without CQDs. This enhancement is attributed to the ability of CQDs to mitigate structural degradation and prevent aggregation during repeated cycling. The rate capability also improved, with the CQD-modified composite demonstrating higher capacity retention at 10 A g^−1^, indicating superior electrochemical performance at high discharge rates. This is owing to the enhanced ionic and electronic conductivity provided by the CQDs, which facilitate faster charge transfer kinetics [160]. In supercapacitors, CQDs contribute to increased energy density and improved power delivery by participating in reversible redox reactions, which are facilitated by the presence of functional groups on the CQD surface. Studies on CQD-modified composites revealed a 25% increase in energy density and a 30% enhancement in power density compared with unmodified composites, as measured through CV and galvanostatic charge/discharge testing. The improved performance is linked to the pseudocapacitive behaviour of CQDs, which adds Faradaic charge storage mechanisms to the electric double-layer capacitance. This electron storage capability enables electrochemical devices to maintain stable, high-rate performance over extended cycles, making CQD-enhanced composites highly suitable for high-performance applications such as energy storage systems and advanced sensors [161].

Furthermore, the integration of CQDs improves the stability and durability of electrochemical devices. They contribute to the structural integrity of the composite, ensuring that the CFs remain stable during long-term cycling and preventing issues such as electrode degradation, electrolyte leakage and capacity fading. This results in a durable, stable composite, crucial for long-lasting performance in energy storage and electrochemical applications. CQDs significantly enhanced the mechanical and electrochemical stability of CFCs, reducing capacity loss and extending the cycling lifetime of the devices. The composites incorporating CQDs demonstrated a 12% lower resistance to charge/discharge cycles, highlighting their improved electrochemical stability over time. Additionally, CQDs have proved effective in detecting metal ions and organic pollutants in water [162]. CFCs functionalized with CQDs were applied for the detection of hexavalent chromium (Cr (VI)) in aqueous solutions. The CQDs enhanced the sensitivity and selectivity of the composite, allowing it to detect low concentrations of Cr (VI) with excellent electrochemical performance. This improvement was attributed to the high surface area and quantum effects of the CQDs, resulting in a detection limit as low as 0.5 µM for Cr (VI). These results demonstrate the potential of CQD-functionalized composites for advanced sensing applications [163].

Table 4 summarizes the electrochemical, triboelectric, thermoelectric and PEC properties of various nanomaterials, highlighting their potential applications in energy conversion and storage. CNTs exhibit high electrical conductivity and triboelectric output, with moderate thermoelectric and PEC effects. Graphene and GO show excellent triboelectric and thermoelectric performance, along with strong piezoelectric responses. Metal oxide nanoparticles, such as MnO_2_, demonstrate high specific capacitance and strong PEC effects but have low thermoelectric performance. Conductive polymers like PPy and PANI offer high triboelectric output and moderate PEC effects owing to their flexibility. Metal nanoparticles, such as gold and silver, exhibit high thermoelectric effects but limited triboelectric and PEC performance. QDs and CQDs show strong PEC effects and moderate triboelectric and thermoelectric properties owing to their tunable band gaps and quantum confinement. SiO₂ NPs and nanoclays have limited triboelectric and thermoelectric effects but can enhance mechanical stability in composites. Nanowires, such as silicon and SnO₂, demonstrate high triboelectric and PEC effects owing to their high surface area and mechanical flexibility. Overall, the table provides a comparative analysis of how different nanomaterials can be used for specific energy-related applications.

**Table 4.** Analysis of nanomaterials in CFCs based on electrochemical, triboelectric, thermoelectric and PEC properties.

nanomaterial	electrochemical parameters	triboelectric effect	thermoelectric effect	piezo-electrochemical effect	ref
**CNTs**	— electrical conductivity: approximately 1000 s cm^−1^ — specific surface area: up to 2630 m² g^−1^ — cycle stability: high rate capability with minimal capacity decay after thousands of cycles	high triboelectric output owing to high surface area and conductivity	moderate thermoelectric effect owing to high electrical conductivity and low thermal conductivity	act as flexible effect but can enhance PEC coupling in composites	[115–118]
**graphene and GO**	— electrical conductivity: approximately 10 000 S m^−1^ — surface area: up to 2630 m² g^−1^ — cycle life: excellent stability over thousands of charge/discharge cycles	excellent triboelectric performance owing to high surface area and mechanical strength	high thermoelectric potential owing to excellent electrical conductivity and tunable thermal properties	strong piezoelectric response for pressure-induced energy conversion	[122–126]
**metal oxide nanoparticles (e.g. MnO_2_, Co_3_O_4_, NiO**)	— specific capacitance: approximately 200–1500 F g^−1^ (MnO_2_) — energy density: approximately 50 Wh kg^−1^ (MnO_2_) — cycle stability: high stability (up to 5000 cycles with <10% degradation)	moderate triboelectric effect owing to surface redox activity and high surface area	low thermoelectric effect owing to high thermal conductivity and moderate electrical conductivity	strong PEC effect owing to redox activity and mechanical deformation under stress	[129–132]
**conductive polymers (e.g. polypyrrole, polyaniline**)	— conductivity: approximately 1000 S cm^−1^ (PANI) — mechanical strength: increased flexibility and mechanical resilience — efficiency: up to 99% charge/discharge efficiency	high triboelectric output owing to flexibility and high surface charge density	low thermoelectric effect owing to moderate electrical conductivity and high thermal conductivity	moderate PEC effect owing to mechanical flexibility and ion mobility	[134–137]
**metal nanoparticles (e.g. gold, silver, copper**)	— catalytic activity: high (50% increase in ORR for Au) — electron transfer: fast (up to 10⁻³ s) — charge/discharge performance: high current density and improved rate capability	low triboelectric effect owing to high conductivity and low surface charge generation	high thermoelectric effect owing to excellent electrical conductivity and low thermal conductivity	limited PEC effect but can enhance catalytic activity under mechanical stress	[139–142]
**quantum dots**	— stability: low degradation over cycles (high stability) — tuning properties: adjustable band gap for specific applications — charge storage: increased charge storage and stability	moderate triboelectric effect owing to tunable surface properties and high surface area	moderate thermoelectric effect owing to tunable band gap and size-dependent properties	strong PEC effect owing to quantum confinement and strain-induced charge separation	[145–148]
**silica nanoparticles**	— surface area: up to 800 m² g^−1^ — stability: high chemical stability, prevents degradation — ion storage: increased storage capacity during charge/discharge	low triboelectric effect owing to insulating nature but can enhance triboelectric composites	very low thermoelectric effect owing to insulating properties	limited PEC effect but can improve mechanical stability in composite materials	[149–152]
**nanowires (e.g. silicon nanowires, tin oxide nanowires**)	— surface area: approximately 2000 m² g^−1^ — mechanical strength: stronger composite material — ion diffusion: enhanced ion mobility, improving power density and cycle stability	high triboelectric effect owing to high surface area and mechanical flexibility	moderate thermoelectric effect owing to high surface area and tunable electrical properties	strong PEC effect owing to mechanical deformation and high surface area for ion interaction	[153–155]
**nanoclays (e.g. montmorillonite**)	— surface area: up to 2000 m² g^−1^ — ion storage: high capacity for intercalation — mechanical integrity: stronger composite material with better mechanical resilience	moderate triboelectric effect owing to high surface area and mechanical resilience	very low thermoelectric effect owing to insulating properties	moderate PEC effect owing to intercalation and mechanical deformation	[157–159]
**carbon quantum dots**	— electron storage: approximately 500 mAh g^−1^ — conductivity: approximately 100 S cm^−1^- electrochemical stability: excellent (up to 5000 cycles with minimal decay)	moderate triboelectric effect owing to high surface charge density and conductivity	moderate thermoelectric effect owing to tunable band gap and size-dependent properties	strong PEC effect owing to quantum confinement and strain-induced charge separation	[21,160,161]

## 9. Sustainability and environmental implications

The integration of CFCs with nanomaterials has revolutionized electrochemical applications, offering unparalleled mechanical strength, electrical conductivity and energy storage capabilities. However, the environmental and sustainability implications of their production, use and disposal present significant challenges that must be addressed to ensure their long-term viability in sustainable energy systems. The energy-intensive nature of CF and nanomaterial production, coupled with difficulties in recycling and potential environmental toxicity, underscores the need for innovative strategies to mitigate their ecological footprint. The production of CFs, primarily from polyacrylonitrile (PAN) precursors, is a major contributor to the environmental impact of CFCs. The process involves high-temperature pyrolysis (1000–3000°C), which consumes substantial energy and generates significant greenhouse gas emissions [54]. For instance, producing 1 kg of PAN-based CFs can emit up to 30−50 kg of CO₂ equivalents, depending on the energy source [141]. Similarly, nanomaterial synthesis, such as graphene and CNTs via CVD or liquid-phase exfoliation, is energy-intensive and often relies on hazardous chemical precursors, further increasing environmental burdens [13]. Metal oxide nanoparticles (e.g. MnO₂, Co₃O₄) and QDs used in CFCs also require resource-intensive processes, including mining and refining of raw materials, which contribute to ecosystem degradation and water pollution [148]. These factors make the large-scale production of CFCs and nanomaterial-enhanced composites environmentally costly, posing barriers to their adoption in sustainable applications like EVs and renewable energy systems.

Recycling and end-of-life management of CFCs represent another critical sustainability challenge. CFs and nanomaterials are inherently non-biodegradable, and their integration into complex polymer matrices complicates disassembly and recycling. Traditional recycling methods, such as mechanical grinding or thermal pyrolysis, often degrade the mechanical and electrochemical properties of recovered CFs, limiting their reuse in high-performance applications [54]. For example, thermal recycling at 500−700°C can reduce CF tensile strength by up to 30%, rendering them unsuitable for structural or electrochemical roles [141]. Moreover, nanomaterials like CNTs, graphene and metal oxides pose unique recycling challenges owing to their nanoscale size and chemical stability. Improper disposal of these materials risks environmental contamination, as heavy metals in QDs or metal oxides (e.g. Co, Ni) can leach into soil and groundwater, causing long-term ecological harm [148]. The lack of standardized recycling protocols for nanomaterial-enhanced CFCs further exacerbates these issues, highlighting the need for sustainable lifecycle management strategies. The potential toxicity of nanomaterials used in CFCs raises additional environmental concerns. CNTs and graphene, while highly effective in enhancing electrochemical performance, have been shown to exhibit cytotoxicity and environmental persistence under certain conditions [141]. For instance, poorly dispersed CNTs can form aggregates that are harmful to aquatic organisms if released into water systems. Similarly, metal oxide nanoparticles, such as MnO₂ and Co₃O₄, used for their pseudocapacitive properties, may dissolve or oxidize in environmental conditions, releasing toxic ions [148]. QDs containing heavy metals (e.g. cadmium, lead) pose significant risks if not properly managed, as their small size enables easy dispersion into ecosystems [145]. These concerns necessitate rigorous assessment of nanomaterial lifecycle impacts, from synthesis to disposal, to prevent unintended environmental consequences.

To address these sustainability challenges, researchers are exploring eco-friendly alternatives and advanced recycling technologies. Bio-based precursors, such as lignin and cellulose, offer a promising alternative to PAN for CF production. Lignin-based CFs require lower processing temperatures (around 800−1200°C) and can reduce CO₂ emissions by up to 50% compared with PAN-based fibres [138]. Similarly, green synthesis methods for nanomaterials are gaining traction. For example, biomass-derived CQDs and GO from agricultural waste reduce reliance on energy-intensive processes and hazardous chemicals [21]. Solvent-free synthesis techniques and electrochemical exfoliation of graphene further minimize environmental impacts while maintaining high material quality [13]. These approaches not only lower the carbon footprint but also enhance the economic feasibility of large-scale CFC production. Advanced recycling technologies are critical for improving the sustainability of CFCs. Chemical recycling methods, such as solvolysis, use supercritical fluids to separate CFs from polymer matrices without significant degradation, preserving up to 90% of their mechanical properties [54]. Microwave-assisted pyrolysis offers an energy-efficient alternative, reducing processing temperatures and energy consumption compared with traditional thermal methods [141]. Additionally, designing recyclable polymer matrices, such as thermoplastic resins, facilitates easier CF recovery and reuse. For nanomaterials, strategies like magnetic separation of metal oxides or enzymatic degradation of CNTs are being investigated to enable selective recovery [148]. These innovations promote a circular economy for CFCs, reducing waste and resource consumption while maintaining performance in electrochemical applications.

## 10. Challenges, advancements and future potential of carbon fibre composites and nanomaterial in electrochemistry

CFCs have gained widespread attention for their superior mechanical properties, electrical conductivity and lightweight nature, making them ideal candidates for electrochemical applications such as energy storage, sensing and catalysis. The integration of nanomaterials into CFCs has opened new avenues to enhance their electrochemical performance, offering improvements in charge/discharge cycles, cycling stability and overall efficiency. However, despite significant advancements, challenges remain that need to be addressed to fully realize their potential in practical applications. This section explores the current challenges, recent advancements and future directions for CFCs, particularly those enhanced by nanomaterials, in electrochemical systems [93,98].

### 10.1. Current challenges in carbon fibre composits and nanomaterial integration

CFCs have demonstrated remarkable potential for a wide range of electrochemical applications, including energy storage, catalysis and sensing. However, one of the biggest challenges facing the widespread use of CFCs is the high cost associated with their production. High-quality CFs, which are essential for ensuring the performance and longevity of CFCs, are expensive to manufacture. These fibres typically require specialized processing, including high temperatures and the use of costly precursor materials such as PAN or pitch, making them more expensive than traditional materials. For instance, PAN-based CFs require pyrolysis at extremely high temperatures (up to 1000−3000°C), adding to the energy and operational costs. When combined with advanced nanomaterials like graphene, CNTs and metal oxide nanoparticles, the production costs increase even further, making it difficult to scale these materials for large-scale industrial applications. The integration of nanomaterials into CFCs is a key factor in enhancing their electrochemical properties, such as conductivity, surface area and ion diffusion [115,138]. However, many of these nanomaterials are also expensive to produce in large quantities. For example, graphene production, particularly in its high-quality form, involves energy-intensive processes such as CVD or liquid-phase exfoliation, both of which are costly. The CVD process, for instance, requires high temperatures and specialized equipment, making it economically unfeasible for large-scale production. Similarly, CNTs and metal oxide nanoparticles often require complex synthesis techniques to ensure high purity and consistency. For example, the growth of CNTs using CVD or laser ablation is not only expensive but also difficult to control on a large scale. This makes the manufacturing process for CFCs that incorporate these nanomaterials prohibitively expensive, limiting their commercialization for large-scale industrial applications [119,120].

In addition to the high material costs, the fabrication processes for CFCs themselves also contribute to the overall expense. The production of CFCs often involves intricate processes, such as layering, curing and moulding, which are labour-intensive and require precise control over material properties to ensure optimal performance. These processes, while essential for achieving the desired electrochemical characteristics, further drive up costs. For example, the manufacturing of CFCs for applications in energy storage devices such as supercapacitors or Li-ion batteries often involves expensive techniques such as chemical vapour infiltration, which adds to the overall cost of production. Additionally, the post-processing steps, including the activation of nanomaterials or functionalization of the composite surface, also incur additional expenses. This complexity in the production process, combined with the need for expensive equipment and controlled environments, makes scaling up production for commercial use challenging. To address these scalability and cost-effectiveness challenges, research efforts are increasingly focused on finding alternative, more affordable materials and processes [130,134]. For instance, efforts are being made to develop cheaper precursor materials for CFs, such as biomass-derived sources or lower-cost PAN precursors, which could reduce the overall production costs. One example is the development of bio-based CFs from lignin, which could lower costs and provide a more sustainable option. Similarly, more affordable nanomaterial production methods are being explored, such as the use of less expensive chemicals for synthesizing GO or employing solution-based methods for CNT growth, which could be more easily scaled up. New manufacturing techniques, such as roll-to-roll processing or scalable CVD systems, are also being researched to improve production efficiency and lower the costs of large-scale fabrication. Moreover, researchers are exploring hybrid materials, such as combining low-cost polymers with nanomaterials or using lower-cost metal oxide nanoparticles, to optimize performance while reducing costs. Achieving cost reductions without sacrificing performance is crucial for enabling the widespread use of CFCs in commercial electrochemical systems, especially in sectors such as EVs, renewable energy storage and large-scale industrial applications. For instance, some companies are now focusing on integrating CFs into lower-cost composite systems that still retain high electrochemical performance but at a fraction of the cost [138,143].

The successful integration of nanomaterials into CFCs plays a crucial role in maximizing their electrochemical performance. However, one of the major challenges in developing high-performance CFCs is ensuring the uniform dispersion of nanomaterials within the CF matrix. Nanomaterials such as CNTs, graphene and metal oxide nanoparticles have unique properties that make them ideal for enhancing conductivity, ion diffusion and overall mechanical strength. However, their incorporation into CFCs can be problematic owing to their tendency to agglomerate, especially at higher concentrations. This aggregation leads to a non-uniform distribution of nanomaterials within the composite, which can negatively affect the overall performance. For example, the agglomeration of CNTs can reduce the surface area available for electron and ion interactions, leading to a decrease in the material’s conductivity and overall electrochemical efficiency. Achieving uniform dispersion of nanomaterials is particularly challenging owing to their intrinsic properties [117,128]. Many nanomaterials, including CNTs and graphene, are highly hydrophobic and have poor solubility in solvents commonly used in composite fabrication processes. This makes it difficult to evenly distribute these materials within the CF matrix. Additionally, the large surface area and strong van der Waals interactions between individual nanoparticles can cause them to aggregate. Without the appropriate dispersion strategy, the nanomaterials may form clusters that disrupt the structure and performance of the composite. For example, in the case of CNTs, if they are not well-dispersed, their inherent properties such as high conductivity and mechanical strength will not be fully realized, leading to a less effective composite material. Moreover, the interface between the nanomaterials and the CF matrix is critical for maintaining the long-term stability and electrochemical properties of the composite. A weak or unstable interface can result in poor adhesion between the nanomaterials and the CFs, leading to reduced mechanical properties and performance degradation over time, especially under repeated charge/discharge cycles. To enhance the electrochemical properties, it is essential that the nanomaterials are well-bonded to the surface of the CFs to prevent them from detaching during cycling, which can result in the loss of electrochemical activity and mechanical strength. Various techniques, such as surface functionalization or chemical modification of the CF surface, can be used to improve the interaction between the nanomaterials and the fibre matrix. For example, functional groups such as carboxyl, hydroxyl or amino groups can be introduced on the surface of CNTs or graphene, which can help improve their dispersion and compatibility with the CF matrix [18,120].

Improving the long-term stability of CFCs requires effective dispersion and integration of nanomaterials. Techniques like using surfactants, solvents or sonication (e.g. in dimethyl formamide (DMF) or N-methyl-2-pyrrolidone (NMP)) help break up agglomerates and enhance dispersion of nanomaterials like graphene and CNTs. Functionalized nanomaterials or polymeric binders can further stabilize and distribute these materials within the CF matrix. Additionally, optimizing processing conditions such as temperature, pressure and mixing time improves bonding between the nanomaterials and the CFs. Overcoming challenges in dispersion and integration is critical for enhancing the durability and performance of CFCs, enabling their successful application in energy storage, sensors and other electrochemical systems [64,82].

One of the key challenges for the practical use of CFCs in electrochemical systems is their long-term stability, particularly in energy storage devices like supercapacitors and batteries. These devices must maintain both structural integrity and electrochemical performance over prolonged use, especially during repeated charge/discharge cycles. Operating in harsh environments exposes CFCs to electrical and chemical stresses, which over time can cause material degradation, reduced efficiency and shorter lifespans. This is a critical issue for large-scale applications like EVs, grid energy storage and portable electronics. Nanomaterials integrated into CFCs, such as CNTs, graphene and metal oxide nanoparticles, are crucial for enhancing conductivity, charge storage and mechanical strength [95,141]. However, their durability is tested under repeated cycling, which exposes them to extreme conditions like high voltages, temperature fluctuations and electrolyte interactions. This exposure can lead to the degradation of the nanomaterials, including oxidation or dissolution of metal oxides, resulting in a loss of electrochemical activity. For instance, metal oxide nanoparticles like MnO_2_ and Co_3_O_4_, commonly used for their pseudocapacitive properties, can experience structural collapse and reduced surface area after extensive cycling, severely impacting their energy storage capabilities [133,148].

To improve the long-term stability of CFCs, enhancing the nanomaterials' robustness and their integration with the CF matrix is crucial. Surface modifications, such as coating metal oxide nanoparticles or using dopants, can increase resistance to oxidation and dissolution, improving durability. The interface between the nanomaterials and the fibres is also key, as a weak bond can cause detachment and accelerate degradation. Strong adhesion is vital for maintaining mechanical strength and electrochemical properties. Incorporating stabilizing agents like conductive polymers (e.g. PPy, PANI) or silica nanoparticles can further enhance stability, prevent oxidation and provide additional protection. To assess long-term stability, accelerated aging tests and EIS are essential. By addressing these factors, researchers can develop more durable and efficient CFCs for energy storage applications [136,152].

The growing use of CFCs in electrochemical applications raises concerns about their environmental impact. The production of CFCs, especially those with nanomaterials like CNTs, graphene and metal oxide nanoparticles, is energy-intensive and involves processes like pyrolysis and CVD, which contributes to carbon emissions and pollution. Additionally, these materials are difficult to recycle owing to their non-biodegradability and complex structure. Nanomaterials such as CNTs and graphene complicate the recycling process and, if improperly disposed of, could leach into ecosystems and cause environmental harm, particularly when toxic metals are involved. Metal oxide nanoparticles like MnO_₂_, Co_₃_O_₄_ and NiO in CFCs boost energy storage performance but raise environmental concerns, as improper disposal can lead to contamination. QDs with heavy metals also pose risks. To mitigate this, researchers are developing bio-based CFCs using sustainable materials like lignin and biodegradable nanomaterials. Advances in recycling technologies, such as chemical recycling, are also being explored to reduce the environmental impact of CFCs while maintaining performance [141,148].

### 10.2. Recent advancements in carbon fibre composites and nanomaterial integration

Recent advancements in nanomaterial integration into CFCs have significantly enhanced their electrochemical performance, paving the way for improved energy storage devices and sensors. The incorporation of nanomaterials such as graphene, CNTs, metal oxide nanoparticles and conductive polymers has led to substantial improvements in properties such as electrical conductivity, surface area, charge storage capacity and cycling stability. These enhancements are crucial for optimizing the performance of supercapacitors, batteries and electrochemical sensors, making them more efficient and durable in real-world applications. Graphene and CNTs, both renowned for their exceptional electrical conductivity and high surface areas, have been widely used to enhance the electrochemical performance of CFCs. Graphene’s two-dimensional structure provides a large surface area for ion adsorption, which is essential for improving charge storage in energy devices. Additionally, the high conductivity of graphene facilitates efficient electron transfer, which is vital for high-rate charge/discharge cycles in supercapacitors and batteries. Similarly, CNTs, with their one-dimensional structure, offer an excellent pathway for electron flow, while also providing a large surface area that increases the number of active sites for ion storage. This combination of conductivity and surface area allows for better energy storage and faster charge/discharge times in electrochemical applications [103,113].

Metal oxide nanoparticles have also demonstrated significant improvements in the electrochemical performance of CFCs. These materials exhibit pseudocapacitive behaviour, where charge storage occurs through faradaic processes in addition to the electrical double-layer capacitance of the CFs. MnO_2_, in particular, is known for its high specific capacitance, and its integration into CFCs leads to improved energy density and long-term cycling stability in supercapacitors. Co_3_O_4_ has shown similar benefits, enhancing the charge storage capacity and stability of CFCs. The ability of these metal oxides to undergo redox reactions during charge/discharge cycles boosts the overall efficiency and increases the energy storage capacity of the composites [142]. Conductive polymers, such as PPy and PANI, have further improved the electrochemical performance of CFCs. These polymers not only provide additional conductivity but also contribute to the mechanical properties of the composite, allowing for enhanced flexibility and structural integrity. In energy storage devices like supercapacitors, PPy and PANI can enhance the overall charge storage capacity and cycling performance by improving the ion transport and providing additional pathways for electron movement. Their incorporation into CFCs leads to an increase in both the capacitance and the overall performance of energy devices, making them more efficient in applications such as flexible electronics and wearable energy storage systems. These advancements in nanomaterial integration have significantly pushed the boundaries of what CFCs can achieve in electrochemical systems. By enhancing key properties such as electrical conductivity, surface area, charge storage capacity and cycling stability, these materials have unlocked new possibilities for high-performance energy storage devices, sensors and other electrochemical applications. As research continues in this field, further innovations in nanomaterial integration and composite design will probably lead to even greater enhancements in the electrochemical performance of CFCs, making them more suitable for large-scale industrial applications [69,137].

Hybrid nanomaterial composites, combining multiple nanomaterials with CF matrices, significantly enhance the electrochemical performance of CFCs. By using the synergistic effects of conductive polymers, metal oxide nanoparticles, and carbon-based nanostructures, these composites optimize charge storage, conductivity, mechanical strength and flexibility. A notable example is the combination of conductive polymers like PPy or PANI with metal oxides such as MnO₂ or Co₃O₄. The polymers improve conductivity and ion storage, while the metal oxides contribute pseudocapacitive behaviour, boosting energy density and charge storage. This hybrid approach enhances the mechanical strength and flexibility, making it suitable for flexible electronics and wearable energy storage devices, like supercapacitors. Hybrid composites combining CNTs or graphene with metal oxide nanoparticles offer enhanced electrochemical performance. CNTs and graphene provide excellent conductivity and high surface area, facilitating electron transfer, while metal oxides increase charge storage and pseudocapacitive behaviour. This synergy results in composites with higher energy densities and improved cycling stability, as seen in Li-ion batteries with CNTs and Co_3_O_4_. Additionally, hybrid composites incorporating silica nanoparticles and conductive polymers like PPy enhance electrochemical stability, conductivity and durability, making them ideal for energy storage devices and sensors. These advancements offer a promising route to optimizing energy storage and electrochemical applications [113,127,132].

The integration of nanomaterials into CFCs has significantly enhanced the development of flexible and wearable electrochemical devices, opening new possibilities in wearable electronics and healthcare monitoring. These devices, including flexible batteries, supercapacitors and sensors, benefit from the unique combination of CFs' mechanical flexibility and the enhanced electrochemical properties of nanomaterials. This synergy enables the creation of lightweight, conformable devices with high electrochemical performance, ideal for integration into smart clothing, wearable sensors and flexible energy storage systems. CFs are inherently strong, lightweight and flexible, making them perfect for electrochemical devices that must bend, stretch or flex without compromising performance. When combined with nanomaterials like graphene, CNTs and conductive polymers, these composites experience enhanced electrical conductivity, high surface area and superior charge storage capabilities. Graphene and CNTs facilitate faster electron transfer, while conductive polymers like PANI and PPy contribute flexibility and ion storage capacity. The result is the development of flexible supercapacitors with high energy and power densities, making them ideal for wearable applications where both mechanical flexibility and high performance are required [128,137].

The integration of nanomaterials such as metal oxide nanoparticles and QDs into CFCs has greatly enhanced the performance of wearable sensors. These sensors offer increased sensitivity, rapid response times and the ability to detect a wide range of biomolecules and environmental parameters, making them invaluable in healthcare for monitoring physiological signals like glucose, heart rate and body temperature. The flexibility and improved electrochemical response of these composites allow for seamless integration into clothing or skin patches, offering continuous and comfortable health monitoring. In energy storage, nanomaterial-enhanced CFCs have led to the development of flexible batteries and supercapacitors with superior charge storage and cycling stability. Flexible power sources, which can be incorporated into wearable devices like fitness trackers, smartwatches and health sensors, provide a reliable energy supply without added weight or stiffness [140]. These advancements in wearable electrochemical devices are paving the way for more efficient, durable and adaptable healthcare and energy solutions. As research progresses, these devices will become even more integrated into daily life, offering greater functionality in wearable electronics and healthcare monitoring.

Advances in fabrication techniques have significantly improved the production and performance of CFCs integrated with nanomaterials. Techniques like electrospinning, CVD and sol–gel methods offer precise control over nanomaterial structure, dispersion and alignment, crucial for enhancing electrochemical properties, conductivity, mechanical strength and overall performance. Electrospinning, in particular, allows for the creation of ultrathin fibres with uniform nanomaterial distribution, enhancing surface area and conductivity. This method is ideal for applications in wearable electronics, sensors and energy storage devices, providing higher power densities and faster charge/discharge rates. CVD and sol–gel methods are key techniques for fabricating CFCs with nanomaterials. In CVD, gaseous precursors deposit nanomaterials like graphene or CNTs directly onto CFs, enhancing conductivity, electrochemical performance and mechanical properties. This process is ideal for high-performance electrodes in batteries, supercapacitors and fuel cells. Sol–gel methods use metal alkoxides or salts to form a gel, allowing controlled deposition of metal oxide nanoparticles (e.g. MnO_2_, Co_3_O_4_, TiO_2_) on CFs, improving charge storage capacity and cycling stability. Both methods enable scalable production of high-performance CFCs with enhanced electrochemical properties, benefiting energy storage, sensors and wearable electronics. As fabrication techniques evolve, the potential for more efficient methods will further enhance CFCs' properties and expand their applications [129,139].

### 10.3. Future potential and applications of carbon fibre composites and nanomaterial in electrochemistry

In order to fully realize the potential of CFCs in electrochemical systems, one of the most pressing challenges is the reduction of production costs and the scaling up of manufacturing processes. While CFCs offer remarkable electrochemical properties, such as enhanced conductivity, high surface area and mechanical strength, the high cost of CFs and nanomaterials significantly limits their large-scale industrial application. To make CFCs more accessible for widespread use, particularly in energy storage devices, sensors and wearable electronics, innovations in scalable manufacturing methods and cost-effective nanomaterial synthesis must be prioritized. Nanomaterial synthesis plays a crucial role in the cost structure of CFCs. Nanomaterials such as graphene, CNTs and metal oxide nanoparticles are essential for enhancing the electrochemical properties of CFCs. However, current synthesis methods for these materials, such as CVD and chemical exfoliation, can be expensive, energy-intensive and difficult to scale up. Researchers are actively exploring more cost-effective and scalable alternatives, such as the use of low-cost precursors, solvent-free processes and green synthesis techniques [53,101]. For example, the use of natural sources for graphene or carbon nanotube production, or simpler sol–gel processes for metal oxide nanoparticles, can significantly reduce costs while still achieving the desired material properties. The development of methods that allow for the continuous production of high-quality nanomaterials is crucial for making CFCs more economically viable for large-scale manufacturing [13].

Optimization of manufacturing processes for CFCs is equally important in achieving scalability and cost reduction. Traditional methods of CF production, such as PAN precursor-based processes, are energy-intensive and expensive. In addition, incorporating nanomaterials into CFs typically requires complex fabrication techniques, such as electrospinning, dip-coating or chemical vapour deposition, which can further increase the production cost. Innovations in manufacturing processes that allow for seamless integration of nanomaterials into CFCs during production, such as *in situ* polymerization or direct growth of nanomaterials on CFs, could significantly reduce costs and improve scalability. Furthermore, optimizing the efficiency of these processes through automation, improved precursors and lower energy consumption would help to make large-scale production of CFCs more economically feasible [136]. The large-scale application of CFCs in electrochemical systems will depend on developing scalable production methods. Industries like EVs, renewable energy and consumer electronics require high-performance, cost-efficient materials. Scalable techniques such as roll-to-roll processing, spray-coating and extrusion could enable mass production while maintaining consistent quality. These methods offer high throughput and lower costs, making CFCs suitable for commercial use in batteries, supercapacitors and flexible electronics. Addressing the cost and scalability of production is crucial to fully unlocking CFCs' potential in electrochemical applications [32].

In the rapidly evolving field of CFCs for electrochemical systems, improving nanomaterial integration is a major focus. One of the key challenges in advancing CFCs is ensuring that nanomaterials such as graphene, CNTs, metal oxides and conductive polymers are uniformly dispersed within the CF matrix. Achieving efficient dispersion not only enhances the electrochemical properties but also ensures that the composites are mechanically robust and stable over long-term use. For example, GO and reduced GO are frequently incorporated into CFCs for their excellent conductivity and high surface area. However, these materials tend to agglomerate owing to their inherent hydrophilicity, which can hinder effective integration. To address this, researchers have developed strategies such as surface functionalization, which can improve the compatibility between GO and CFs. By introducing functional groups (e.g. -OH, -COOH) on the graphene surface, the bonding between the graphene and the CF can be improved, allowing for better load transfer and higher electrochemical performance [122,128].

CNTs, known for their excellent mechanical strength and conductivity, are often added to CFCs to enhance their electrochemical properties. However, CNTs also suffer from agglomeration owing to van der Waals forces, which can negatively impact the overall performance of the composite. To improve the dispersion of CNTs, researchers have explored using surfactants and polymeric coatings that help in dispersing the CNTs evenly across the matrix. For example, polyvinyl alcohol and polyvinylpyrrolidone are often used to disperse CNTs in aqueous solutions before they are incorporated into the CF matrix. In addition to graphene and CNTs, metal oxide nanoparticles (such as MnO_2_, Co_₃_O_₄_ and NiO) are also incorporated into CFCs for pseudocapacitive behaviour, which enhances energy density and cycling stability in supercapacitors. These metal oxides, when integrated with CFs, act as active sites for ion adsorption during charge/discharge cycles. For instance, MnO_₂_ nanoparticles are known to exhibit high pseudocapacitive performance, but their poor conductivity can limit their performance. To overcome this, they are often hybridized with conductive nanomaterials like CNTs or graphene to form hybrid composites that exhibit superior electrochemical performance. Another example is conductive polymers, such as PPy and PANI, which are often used in combination with CFs to enhance charge storage and cycling stability. These polymers can undergo redox reactions during electrochemical processes, which improve the energy storage capacity of the composite [78,129,143]. However, the mechanical properties of conductive polymers can be limited when used alone. To address this, hybrid composites combining conductive polymers with graphene or metal oxide nanoparticles have been developed, showing enhanced flexibility, mechanical strength and electrochemical performance.

A promising advancement in nanomaterial integration is the development of hybrid nanomaterial composites, which combine multiple types of nanomaterials into a single CFC. For instance, combining CNTs with GO enhances both conductivity and surface area, while incorporating metal oxide nanoparticles like Co_₃_O_₄_ and PANI combines the high energy storage capacity of metal oxides with the conductivity and flexibility of CNTs and graphene. These hybrid composites exhibit superior performance in energy storage devices and sensors owing to their synergistic effects. By improving nanomaterial integration through advanced dispersion methods, surface functionalization and hybrid material design, the electrochemical performance and long-term stability of CFCs will be significantly enhanced. Overcoming these integration challenges will unlock the full potential of CFCs in energy storage, sensors and other electrochemical applications, supporting future technological innovations. As the world transitions to renewable energy sources and EVs, the need for high-performance energy storage systems is growing. CFCs, enhanced with various nanomaterials, offer a promising solution owing to their exceptional properties [56,63]. These composites provide superior electrochemical performance, with high energy densities, fast charge/discharge cycles and longer lifespans compared with traditional materials like metals or pure CFs. Nanomaterials such as graphene, CNTs, metal oxide nanoparticles and conductive polymers significantly improve the properties of CFCs. Graphene’s high surface area and electrical conductivity enhance charge storage and facilitate rapid electron transfer, while CNTs offer exceptional mechanical strength and conductivity, ensuring the long-term stability and efficiency of energy storage devices like supercapacitors and Li-ion batteries [69].

Metal oxide nanoparticles, such as MnO₂, Co₃O₄ and NiO, are commonly used in supercapacitors and batteries owing to their ability to promote pseudocapacitive behaviour. These metal oxides enhance energy density and cycling stability by providing additional sites for ion storage and facilitating faster ion diffusion during charge/discharge cycles. For example, MnO₂ nanoparticles have been integrated into CFCs to improve energy density and increase the number of charge/discharge cycles, which is essential for the performance of supercapacitors used in EVs and grid energy storage. The hybridization of nanomaterials in CFCs has further pushed the boundaries of energy storage systems. Combining graphene, CNTs and metal oxide nanoparticles into a single composite material can use the strengths of each material [96,128]. The graphene provides excellent conductivity, while the CNTs offer mechanical reinforcement, and the metal oxides contribute to high-energy storage and long-term cycling stability. These hybrid nanocomposite structures are particularly beneficial in applications where high-performance storage systems are required, such as in EVs, solar energy storage and renewable energy systems. Looking ahead, the development of next-generation batteries, supercapacitors and hybrid storage systems will hinge on advances in nanomaterial design and composite optimization. Nanomaterials will be tailored to enhance charge capacity, conductivity and cycling stability, overcoming challenges like slow charge/discharge rates, limited cycle life and high manufacturing costs. Scalable manufacturing processes for CFCs with nanomaterials will be essential for producing these advanced energy storage systems efficiently and affordably. Enhanced CFCs have the potential to revolutionize energy storage, offering high energy densities, fast charge/discharge capabilities and durability. The ongoing development of nanomaterials will lead to more efficient, cost-effective and sustainable energy storage solutions, supporting the transition to renewable energy and electric mobility [119,121].

As the demand for CFCs grows, sustainability will be a key focus, addressing the environmental impact of production and end-of-life disposal. Current CF production is energy-intensive, generating significant CO₂ emissions, and recycling CFCs remains complex and costly. Innovations in production processes, such as bio-based precursors for CF, biodegradable resins and green chemistry approaches, are being explored to reduce emissions and lower the environmental impact of composite manufacturing. Additionally, improving the energy efficiency of CF production, including the use of renewable energy sources, is essential for the sustainability of these materials. Recycling CFCs presents challenges owing to the difficulty in separating CFs from the polymer matrix, with traditional methods like mechanical grinding and thermal processing having limitations. Advanced techniques such as solvolysis, pyrolysis and microwave-assisted processes are showing promise in improving the recovery of CFs for reuse in new composites. Researchers are also developing recyclable polymer matrices to facilitate easier recovery of CFs, promoting a circular economy for CFCs [54]. The integration of nanomaterials, such as metal nanoparticles, graphene and CNTs, enhances properties but complicates recycling owing to their small size and chemical stability. Designing nanomaterials that can be safely removed reused or biodegraded without leaving harmful residues is crucial for improving the environmental sustainability of these enhanced materials. Furthermore, CFCs are flexible, lightweight and biocompatible, making them ideal for electrochemical sensors used in biomedical applications [115].

## 11. Conclusion

CFCs, enhanced with nanomaterials, offer significant potential to revolutionize energy storage and harvesting technologies. The integration of nanomaterials such as graphene, CNTs and metal oxides has addressed key limitations of CFs, improving their surface area, charge storage capacity and overall electrochemical performance. These advancements enable CFCs to excel in applications such as SBs, supercapacitors and energy harvesting systems, where multifunctionality, durability and lightweight design are critical. While challenges in production scalability and long-term stability persist, the innovations in nanomaterial integration and advanced manufacturing techniques are paving the way for CFCs to play a crucial role in the development of future sustainable energy systems.

## Data Availability

As this is a review article, no new datasets were generated or analysed. All referenced data are available in the cited literature, with DOIs provided where applicable in the reference list. Supporting information, if any, is accessible through the original publications cited in this manuscript. Supplementary material is available online [164].
